# Identification of eQTLs using different sets of single nucleotide polymorphisms associated with carcass and body composition traits in pigs

**DOI:** 10.1186/s12864-023-09863-8

**Published:** 2024-01-02

**Authors:** Felipe André Oliveira Freitas, Luiz F. Brito, Simara Larissa Fanalli, Janaína Lustosa Gonçales, Bruna Pereira Martins da Silva, Mariah Castro Durval, Fernanda Nery Ciconello, Camila Sabino de Oliveira, Lucas Echevarria Nascimento, Izally Carvalho Gervásio, Julia Dezen Gomes, Gabriel Costa Monteiro Moreira, Bárbara Silva-Vignato, Luiz Lehmann Coutinho, Vivian Vezzoni de Almeida, Aline Silva Mello Cesar

**Affiliations:** 1 Luiz de Queiroz College of Agriculture, University of São Paulo, Piracicaba, 13416-000 SP Brazil; 2 Department of Animal Sciences, Purdue University, West Lafayette, IN 47907 USA; 3 Faculty of Animal Science and Food Engineering, University of São Paulo, Pirassununga, 13635- 900 SP Brazil; 4 Unit of Animal Genomics, GIGA-R and Faculty of Veterinary Medicine, University of Liège (B34), Liège, 4000 Belgium; 5 College of Veterinary Medicine and Animal Science, Federal University of Goiás, Goiânia, 74001-970 GO Brazil

**Keywords:** *Sus scrofa*, Expression quantitative trait loci, Transcriptomic, LD pruning

## Abstract

**Background:**

Mapping expression quantitative trait loci (eQTLs) in skeletal muscle tissue in pigs is crucial for understanding the relationship between genetic variation and phenotypic expression of carcass traits in meat animals. Therefore, the primary objective of this study was to evaluate the impact of different sets of single nucleotide polymorphisms (SNP), including scenarios removing SNPs pruned for linkage disequilibrium (LD) and SNPs derived from SNP chip arrays and RNA-seq data from liver, brain, and skeletal muscle tissues, on the identification of eQTLs in the *Longissimus lumborum* tissue, associated with carcass and body composition traits in Large White pigs. The SNPs identified from muscle mRNA were combined with SNPs identified in the brain and liver tissue transcriptomes, as well as SNPs from the GGP Porcine 50 K SNP chip array. Cis- and trans-eQTLs were identified based on the skeletal muscle gene expression level, followed by functional genomic analyses and statistical associations with carcass and body composition traits in Large White pigs.

**Results:**

The number of cis- and trans-eQTLs identified across different sets of SNPs (scenarios) ranged from 261 to 2,539 and from 29 to 13,721, respectively. Furthermore, 6,180 genes were modulated by eQTLs in at least one of the scenarios evaluated. The eQTLs identified were not significantly associated with carcass and body composition traits but were significantly enriched for many traits in the “Meat and Carcass” type QTL. The scenarios with the highest number of cis- (*n* = 304) and trans- (*n* = 5,993) modulated genes were the unpruned and LD-pruned SNP set scenarios identified from the muscle transcriptome. These genes include 84 transcription factor coding genes.

**Conclusions:**

After LD pruning, the set of SNPs identified based on the transcriptome of the skeletal muscle tissue of pigs resulted in the highest number of genes modulated by eQTLs. Most eQTLs are of the trans type and are associated with genes influencing complex traits in pigs, such as transcription factors and enhancers. Furthermore, the incorporation of SNPs from other genomic regions to the set of SNPs identified in the porcine skeletal muscle transcriptome contributed to the identification of eQTLs that had not been identified based on the porcine skeletal muscle transcriptome alone.

**Supplementary Information:**

The online version contains supplementary material available at 10.1186/s12864-023-09863-8.

## Background

 Developing effective breeding strategies and genetic improvement programs are paramount for improving the long-term sustainability of livestock production. In this context, there is a need to determine the impact of genomic variants on gene expression and phenotypic variability related to production and environmental efficiency traits, such as feed efficiency, carcass yield, live weight, and body composition [1]. Genome-wide association studies (GWAS) based on single nucleotide polymorphism (SNP) information and traits related to production efficiency and meat quality traits have been extensively explored in recent years [2, 3]. These studies have contributed to the understanding of the genetic architecture of complex traits in pigs, but most studies were primarily done based on SNPs located in intronic and intergenic regions. Therefore, the use of SNPs obtained from transcriptome sequencing could provide additional information about the SNPs located in transcribed regions of the genome, which have a greater likelihood of being more functionally relevant with greater influence on the phenotypic expression of complex traits [4–6].

Genetic markers (e.g., SNPs) located within coding regions of the genome are more likely to change the level of global gene expression in the most diverse tissues of living organisms. For example, a missense variant could result in the alteration of a codon that encodes a certain amino acid and, consequently, lead to changes in protein synthesis and in the functionality of these proteins in various tissues and physiological processes of organisms [7, 8]. When a SNP is in the promoter region of a gene or 3 prime untranslated region (3’UTR), it can alter the level of gene expression and affect post-transcriptional regulations [7]. Thus, these variants may result in phenotypic differences (e.g., carcass trait, body composition) among individuals in a population.

Due to the reduced genetic variability in livestock populations, SNPs located throughout the genome are in moderate to high linkage disequilibrium (LD) [9–11] and, therefore, could have similar effects on a given trait. So, it is a common practice to perform SNP or tag-SNP pruning based on LD thresholds to eliminate SNPs capturing similar quantitative trait loci (QTL) effects in GWAS, in which only one representative SNP of each LD block is maintained to reduce the total number of statistical tests performed [7, 12–15]. Not performing LD pruning could result in more false positives and decrease the statistical power of the analyses [16–18]. The SNPs from the transcriptome could be in greater proximity and, therefore, in greater LD among themselves. Thus, the level of LD among the studied variants is an important element to be considered in expression QTL (eQTL) identification studies based on transcriptome sequencing data [17, 19, 20].

The integration of SNPs from transcriptome sequencing data from different tissues (e.g., skeletal muscle, liver, brain) with other data sources such as SNP chip arrays (e.g., GGP-50 K genotyping) can provide complementary information about genomic variability related to gene expression in specific tissues such as the skeletal muscle – a key tissue for pork production. The combination of SNPs obtained through sequencing of the RNA from different biological tissues and data sources (i.e., sequencing, genotyping) could enable a more accurate identification of eQTLs that would not be detected by analyzing variants from the skeletal muscle tissue alone. In addition to data integration, it is important to evaluate alternative statistical approaches, such as LD pruning and quality control parameters (e.g., minor allele frequency, genotyping call rate, and variants with extreme departure from the Hardy-Weinberg equilibrium expectations), to adjust the initial data structure and reduce potential biases in the results due to the presence of closely linked or low-quality variants [9, 12, 17, 21–24].

We hypothesize that different combinations of SNPs obtained from alternative biological tissues (e.g., skeletal muscle, liver, and brain) and data sources (GGP-50 K genotyping and RNA-seq) may affect the identification of eQTLs associated with carcass and body composition traits in pigs. Therefore, our primary objectives were to: (1) evaluate the impact of different SNP-set combinations (including LD pruning) derived from SNP chip arrays and RNA-seq data from liver, brain, and skeletal muscle tissues on the identification of eQTLs associated with carcass and body composition traits in Large White pigs; and, (2) investigate candidate genes and biological processes associated with the phenotypic expression of these traits. The phenotypic traits evaluated in this study were slaughter weight (SW; in kg), cold carcass yield as a percentage of the slaughter weight (CCY, in %), loin eye area measured by ultrasound (LEA; in cm²), backfat thickness measured by ultrasound (BFT; in cm), and intramuscular fat content in percentage (IMF, in %).

## Results

### Phenotypes, genotypes, and scenarios

The descriptive statistics of the phenotypic traits evaluated in the study are presented in Table 1 and were previously described by Almeida et al. [25].

**Table 1:** Descriptive statistics of the traits included in the association studies, which were partially described by Almeida et al. [24]

TRAIT	N	Mean	Minimum	Maximum	CV	SD
SW (kg)	72	132.7	107.0	160.0	8.24	10.93
CCY (%)	72	69.9	66.4	73.0	1.76	1.23
LEA (cm^2^)	72	44.3	23.4	57.2	11.69	5.17
BFT (cm)	72	14.7	9.9	23.1	17.21	2.53
IMF (%)	72	2.6	0.2	8.4	52.26	1.23

*SW (kg) *Slaughter weight in kg, *CCY (%) *Cold carcass yield as a percentage of the slaughter weight, *LEA (cm²) *Ultrasound-based loin eye area measured between the 10^th^ and 11^th^ ribs, *BFT (cm) *Backfat thickness measured by ultrasound at the 10^th^ rib, *IMF (%) *Intramuscular fat content in percentage, *N *Number of records, *CV (%) *Coefficient of variation, *SD *Phenotypic standard deviation

The SNPs analyzed in this study were derived from RNA-seq data from brain, liver, and skeletal muscle tissues from 72 pigs and from the genotyping of these same animals with the GeneSeek Genomic Profiler Porcine 50 K (GGP-50 K) SNP chip array. A total of 50,697 SNPs were obtained from the GGP-50 K SNP chip array as well as 2,650,720, 1,816,600, and 4,404,053 SNPs (before quality control) obtained from RNA-seq data of skeletal muscle (*Longissimus lumborum*), liver (right lobe of the liver), and brain (a portion of the middle region of the frontal lobe) tissues, respectively, of the same 72 Large White pigs.

The quality control used for filtering out the SNPs identified from the RNA-seq data considered a *Phred* score (QUAL) equal or greater than 30 (QUAL ≥ 30) and coverage depth (DP) equal or greater than 10 (DP ≥ 10). Only bi-allelic variants from the *Sus scrofa* autosomal chromosomes (SSC) SSC1 to SSC18 were included in further analyses. Thus, 1,609,081, 915,828, and 2,649,856 SNPs from skeletal muscle, liver, and brain tissues, respectively, remained in the dataset for further analyses. Additional quality control filters included removing SNPs with minor allele frequency (MAF) lower than 5%, variants with genotyping rate lower than 95% (more than 5% missing), and extreme departure from Hardy-Weinberg equilibrium (HWE; *p*-value lower than 10^−6^). After that the quality control, 74,812, 50,932, and 117,330 SNPs from the skeletal muscle, liver, and brain tissues, respectively, and 30,872 SNPs from the GGP-50 K array from 72 animals were available for further analyses. A total of 15,090 genes were expressed in the skeletal muscle tissue of the 72 animals, which were normalized and represented as transcripts per million (TPM). The gene expression level was also normalized before fitting the linear models.

All the SNP datasets were combined for the identification of cis- and trans-eQTLs in the skeletal muscle tissue. For that, we considered the scenario with only the SNPs found in the skeletal muscle transcriptome as the base scenario, and subsequently, added the SNPs from the brain and liver transcriptomes and from the 50 K SNP chip array. Hence, the SNPs from the RNA-seq data of the brain and liver tissues and the SNPs from the 50 K SNP chip panel were used alone or combined with the SNPs from the skeletal muscle, which resulted in four scenarios: (S1) only the SNPs from the GGP-50 K; (S2) SNPs from the RNA-seq data of the skeletal muscle (baseline scenario); (S3) SNPs from the GGP-50 K plus the SNPs of the RNA-seq data of the skeletal muscle; (S4) SNPs from the GGP-50 K plus the SNPs of RNA-seq data of the skeletal muscle, liver, and brain tissues. Subsequently, the SNP sets from the four scenarios were LD pruned considering an r² threshold of 0.70, which resulted in four additional scenarios: (S5) SNPs from the GGP-50 K after LD pruning; (S6) SNPs from the RNA-seq data of the skeletal muscle after LD pruning; (S7) SNPs from the GGP-50 K plus the SNPs from the RNA-seq data of the skeletal muscle after LD pruning; (S8) SNPs from the GGP-50 K plus the SNPs from the RNA-seq data of the skeletal muscle, liver, and brain tissues after LD pruning. The number of SNPs before and after the quality control for all scenarios are described in Table 2. Furthermore, a Venn diagram illustrating the scenarios is presented in Additional file 1.

**Table 2:** Number of single nucleotide polymorphisms (SNPs) before and after the quality control for each of the scenarios evaluated

Dataset	Number of SNPs (before quality control)	Number of SNPs after the quality control and prior to LD pruning	Number of SNPs after LD pruning
SNPs from the GGP-50 K SNP chip array	50,697	30,872 (S1)	9,210 (S5)
SNPs from the RNA-seq data of the skeletal muscle	2,591,269	74,812 (S2)	18,933 (S6)
SNPs from the GGP-50 K SNP chip array plus the SNPs from the RNA-seq data of the skeletal muscle	2,701,417	104,699 (S3)	30,037 (S7)
Percentage of SNPs from the RNA-seq or GGP-50 K SNP chip array, respectively		71% | 29%	64% | 36%
SNPs from the GGP-50 K SNP chip array plus the SNPs from the RNA-seq data of the skeletal muscle, liver, and brain tissues	6,675,049	135,996 (S4)	105,870 (S8)
Percentage of SNPs from the RNA-seq or GGP-50 K SNP chip array, respectively	78% | 22%	89% | 11%	

*GGP-50K *SNPs from the GeneSeek Genomic Porcine 50K medium density genotyping array, *LD *Linkage disequilibrium, *RNA-seq *RNA sequencing, *S1-S8 *Scenarios 1 to 8. The percentage of RNA-seq and GGP-50K SNPs represents the proportion of SNPs from the pig transcriptome and from the GGP-50K in scenarios S4, S6, S7, and S8

### Identification of eQTLs across scenarios

**Fig. 1:**
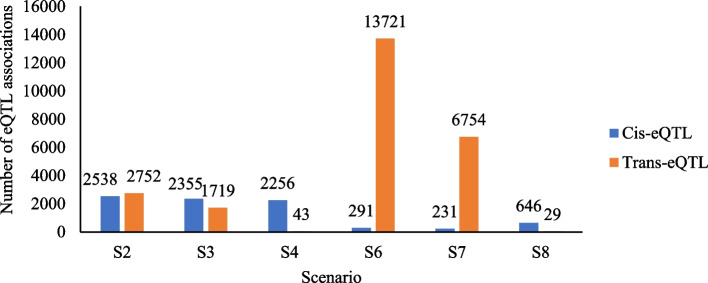
Number of eQTL associations identified for combinations of SNPs pruned and unpruned for linkage disequilibrium (LD). S2: SNPs from the RNA-seq data of the skeletal muscle; S3: SNPs from the GGP-50 K plus the SNPs from the RNA-seq data of the skeletal muscle; S4: SNPs from the GGP-50 K plus the SNPs from the RNA-seq data of the skeletal muscle, liver, and brain tissues; S6: SNPs from the SNPs from the RNA-seq data of the skeletal muscle after LD pruning; S7: SNPs from the GGP-50 K plus the SNPs from the RNA-seq data of the skeletal muscle after LD pruning; and, S8: SNPs from the GGP-50 K plus the SNPs from the RNA-seq data of the skeletal muscle, liver, and brain tissues after LD pruning

 For the cis- and trans-eQTLs identification analyses, genomic windows of up to 1 Mb upstream from the beginning of the regulated gene and 1 Mb downstream from the end of the regulated gene were considered for the cis (local) effect and more than 1 Mb of the regulated gene for the trans (distant) effect. These analyzes were performed for each of the eight scenarios aiming to identify eQTLs based on the gene expression levels in the skeletal muscle tissue. A False Discovery Rate (FDR) of 1% was considered for these analyses. The number of eQTL associations identified were: S1 and S5 = there were no significant cis- or trans-eQTLs; S2: cis-eQTLs = 2,538 and trans-eQTLs = 2,752; S3: cis-eQTLs = 2,355 and trans-eQTLs = 1,719; S4: cis-eQTLs = 2,256 and trans-eQTLs = 43; S6: cis-eQTLs = 291 and trans-eQTLs = 13,721; S7: cis-eQTLs = 231 and trans-eQTLs = 6,754; and, S8: cis-eQTLs = 646 and trans-eQTLs = 29, as shown in Fig. 1.

The number of unique eQTLs (considering a single SNP count) identified in scenarios S2, S3, and S4 ranged from 2,066 to 2,247 for cis-eQTLs and 43 to 379 for trans-eQTLs. In the scenarios with LD pruning (S6, S7, and S8), the number of unique cis-eQTLs ranged from 223 to 612 while the number of trans-eQTLs ranged from 29 to 403. In scenarios S2, S3, and S4, the number of genes regulated by cis- and trans-eQTLs ranged from 159 to 304 and from 8 to 1,965, respectively. The number of genes regulated by cis- and trans-eQTLs in scenarios S6, S7, and S8 ranged from 109 to 185 and from 6 to 5,993, respectively. Table 3 shows the overlap between the significant cis- and trans-eQTLs.

**Table 3:** Description of the percentage and number of cis- and trans-eQTLs (expression quantitative trait loci) identified across scenarios represented by different set of SNPs associated with gene expression levels in the skeletal muscle of Large White pigs

SCENARIO		Cis-eQTLs						Trans-eQTLs					
		S2	S3	S4	S6	S7	S8	S2	S3	S4	S6	S7	S8
**Cis-eQTLs**	S2	2,247	100%	57%	100%	99%	59%	27%	26%	0%	18%	22%	0%
	S3	2,061	2,065	56%	11%	100%	58%	25%	24%	0%	15%	19%	0%
	S4	1,184	1,152	2,066	5%	4%	25%	13%	12%	5%	4%	5%	0%
	S6	223	219	95	223	95%	14%	9%	10%	0%	12%	16%	0%
	S7	182	183	81	174	183	13%	7%	8%	0%	9%	13%	0%
	S8	361	352	156	88	82	612	2%	1%	0%	2%	3%	0%
**Trans-eQTLs**	S2	103	96	50	34	27	7	379	100%	60%	30%	46%	69%
	S3	76	71	36	29	23	4	291	291	58%	7%	36%	66%
	S4	0	0	2	0	0	0	26	25	43	7%	32%	31%
	S6	72	60	18	50	37	9	120	3	93	403	94%	14%
	S7	58	49	13	41	34	9	121	94	3	249	264	14%
	S8	0	0	0	0	0	0	20	19	9	4	4	29

*eQTLs *Expression quantitative trait loci, *SNPs *Single nucleotide polymorphisms, *S2 *SNPs from the RNA-seq data of the skeletal muscle, *S3 *SNPs from the GGP-50K plus the SNPs from the RNA-seq data of the skeletal muscle, *S4 *SNPs from the GGP-50K plus the SNPs from the RNA-seq data of the skeletal muscle, liver, and brain tissues; S6: SNPs from the RNA-seq data of the skeletal muscle after linkage disequilibrium (LD) pruning; S7: SNPs from the GGP-50K plus the SNPs from the RNA-seq data of the skeletal muscle after LD pruning, and, S8: SNPs from the GGP-50K plus the SNPs from the RNA-seq data of the skeletal muscle, liver, and brain tissues after LD pruning. The table diagonal represents the number of eQTLs identified, the values above the diagonal indicate the percentage of overlapping eQTLs among the scenarios, and the values below the diagonal represent the number of overlapping eQTLs across datasets

**Fig. 2:**
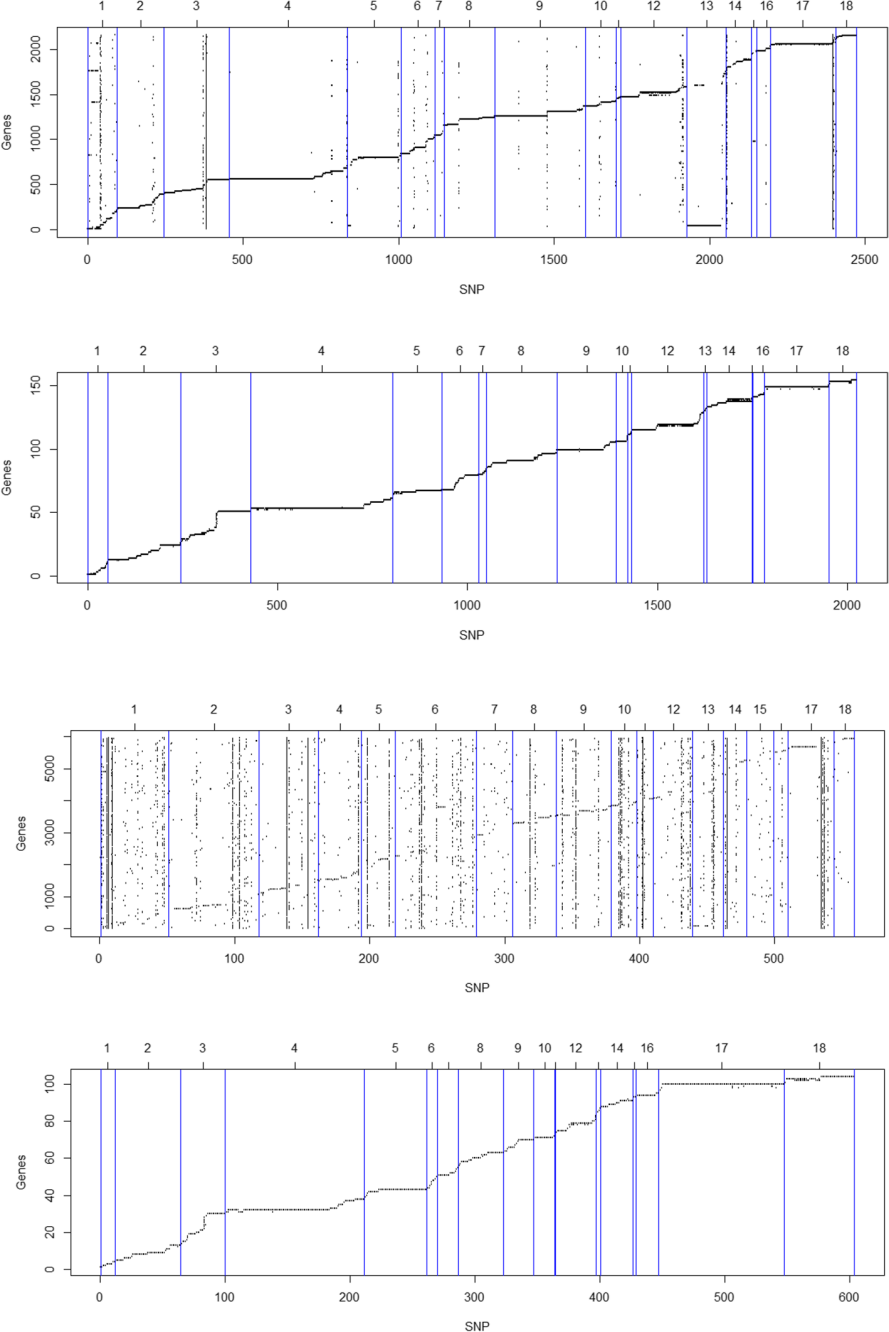
Expression quantitative trait loci (eQTLs) distribution across the autosomal chromosomes for cis- and trans-eQTLs for scenarios S2, S4, S6, and S8 represented by different set of SNPs associated with expression level of skeletal muscle of Large White pigs (Fig. 2a, b and c, and 2d, respectively). The blue lines separate the chromosomes, the Y-axis represents the gene order in relation to chromosome position in the pig genome, and the X-axis represents the SNP order in relation to chromosome position in the pig genome. Figure 2a. S2: SNPs from the RNA-seq data of the skeletal muscle, Fig. 2b. S4: SNPs from the GGP-50 K plus the SNPs from the RNA-seq data of the skeletal muscle, liver, and brain tissues, Fig. 2c. S6: SNPs from the RNA-seq data of the skeletal muscle after linkage disequilibrium pruning, and Fig. 2d. S8: SNPs from the GGP-50 K plus the SNPs from the RNA-seq data of the skeletal muscle, liver, and brain tissues after linkage disequilibrium pruning

The cis- and trans-eQTLs of scenarios S3 and S7 overlapped by 94 to 100% with scenarios S2 and S6. Furthermore, a small number of trans-eQTLs were located on the same chromosome as the modulated gene (S2 = 24, S3 = 15, S4 = 3, S6 = 19, S7 = 13 and S8 = 2). The results of the cis- and trans-eQTLs for all scenarios are presented in Additional file 2. Figure 2a-d illustrate the eQTLs distribution across the autosomal chromosomes for cis- and trans-eQTLs for scenarios S2, S4, S6, and S8, respectively. The diagonal line formed refers to the cis-eQTLs distribution, and the vertical points refer to the trans-eQTLs. The Y-axis represents the gene order in relation to chromosome position in the pig reference genome, and the X-axis represents the SNP order in relation to chromosome position in the pig genome.

## Association of eQTLs with carcass and body composition traits

A total of 2,547, 2,107, 576, and 641 eQTLs (cis- and trans-eQTLs) were identified for the scenarios S2, S4, S6, and S8, respectively. These eQTLs were subsequently used for the association analyses with SW, CCY, LEA, BFT, and IMF. The effects of initial body weight (28.44 ± 2.95 kg) and treatment [basal diet with 1.5% degummed soybean oil, basal diet with 3% soybean oil, basal diet with 3% canola oil and basal diet with 3% fish oil from crooked sardines (*Cetengraulis edentulus*)] were adjusted as continuous covariate and categorical fixed effects, respectively. No significant (FDR < 0.05) or suggestive (0.05 ≤ FDR < 0.10) associations were identified between the eQTLs identified and SW, CCY, LEA, BFT, and IMF for the scenarios S2, S4, S6, and S8. The genomic inflation factor (lambda value - λ) ranged from 0.9 to 1.10, indicating that population structure was properly accounted for in the analyses. The results of the statistical results for the respective scenarios (S1-S8) are presented in Additional file 3.

### eQTLs annotation

**Fig. 3:**
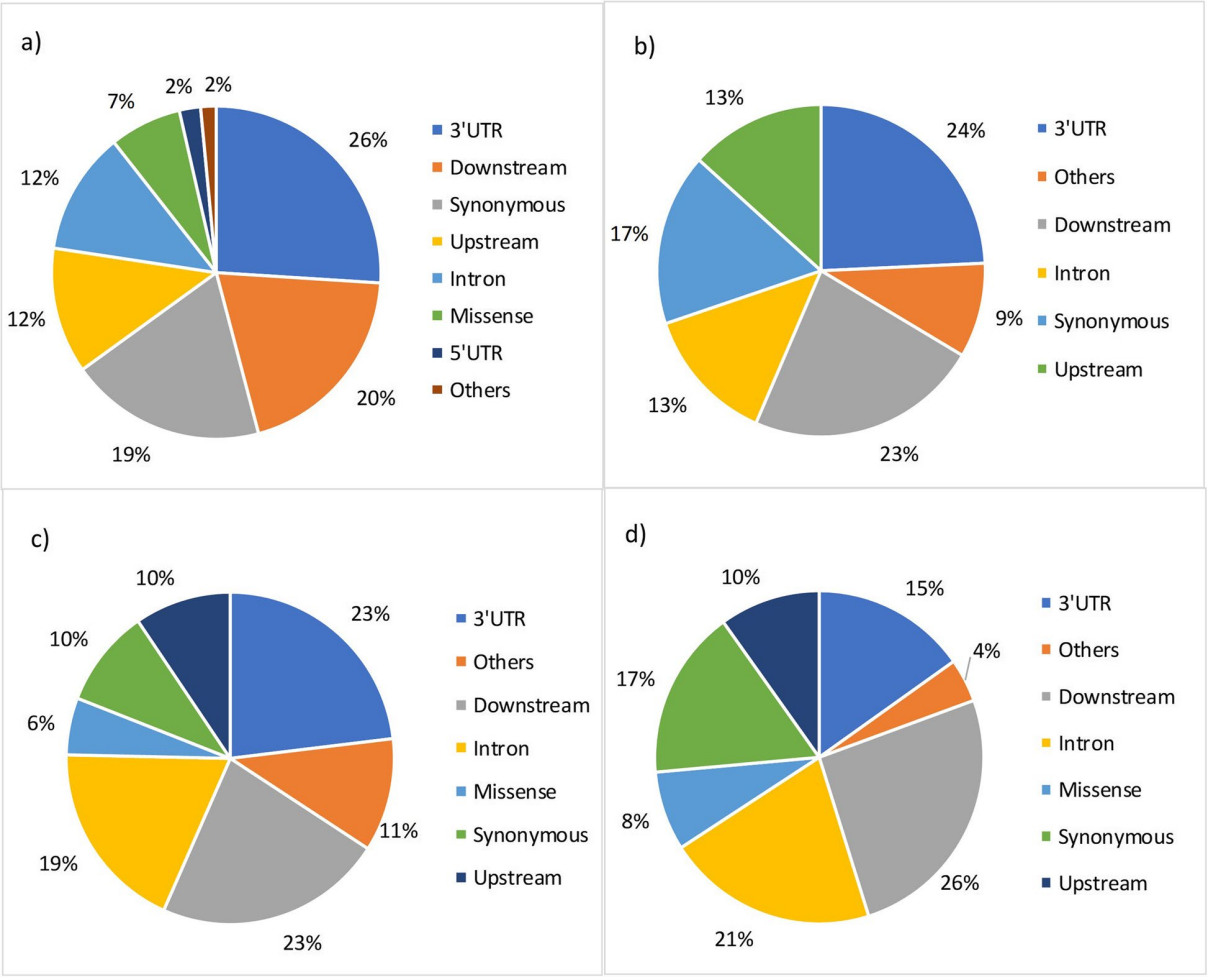
Primary consequences predicted by the Variant Effect Predictor (VEP) tool in scenarios S2 (Fig. 3a), S4 (Fig. 3b), S6 (Fig. 3c), and S8 (Fig. 3d). UTR: untranslated region. UTR variant: a transcript variant that is within an UTR; 3’UTR: an UTR variant of the 3’UTR; 5’UTR: an UTR variant of the 5’UTR; Other: variants non-coding transcript exon, splice polypyrimidine tract, intron intergenic, intron, non-coding transcript, splice region, synonymous, splice acceptor, splice polypyrimidine tract, splice region, intron, splice donor, stop lost, splice donor region, intron, splice polypyrimidine tract, intron, non-coding transcript, splice region, 3’UTR, stop retained, missense, and splice region

For the scenarios S2, S4, S6, and S8, a total of 2,547, 2,107, 576, and 165 variants (cis- and trans-eQTLs) were analyzed, respectively. A total of 1,044 (41.0%), 834 (39.6%), 390 (67.7%), and 68 (41.2%) variants were classified as new variants for scenarios S2, S4, S6, and S8, respectively. Most of these new variants are located within long non-coding RNA (lncRNA) and protein coding genes. Figure 3a-d show the most severe predicted consequences of cis- and trans-eQTLs for each scenario. The Additional file 4 shows the complete Variant Effect Predictor (VEP) annotation for all cis- and trans-eQTLs.

### eQTLs and QTL overlap enrichment analyses

We searched for overlapping genomic position between the eQTLs herein identified and QTL previously reported to be associated with meat and carcass quality and other production traits in pigs using the Genomic Annotation in Livestock for positional candidate LOci (GALLO, [26]) R package. This R package annotates and provides graphical visualization of QTL enrichment analyses. The annotation and enrichment analyses of the eQTLs from each of the scenarios tested (S2, S4, S6, and S8) resulted in 31,023 QTL previously reported in the PigQTLdb database (release 47) [27], considering a window of up to 100 kb downstream and upstream of the genomic coordinates of the cis- and trans-eQTLs. The initial number of SNPs in a single count were 2,247 for cis-eQTLs and 379 for trans-eQTLs in scenario S2; 2,066 of cis-eQTLs and 43 of trans-eQTLs in scenario S4; 223 cis-eQTLs and 403 trans-eQTLs for scenario S6; and 612 cis-eQTLs and 29 trans-eQTLs for scenario S8. The QTLs resulting from the annotation were enriched using a hypergeometric test to reduce potential bias in the results.

For scenarios S2, S4, S6, and S8, the traits “loin muscle area”, “average backfat thickness”, and “abdominal fat weight” from the QTL list of the “Meat and Carcass” type were enriched. The traits “carcass weight (hot)”, “fat-cuts percentage”, “linoleic acid content”, “backfat above muscle dorsi”, “subcutaneous fat area”, and “muscle protein percentage” were also enriched for cis- and trans-eQTLs in scenarios S2 and S6, and only for cis-eQTLs in scenarios S4 and S8. The traits “fat weight (total)” and “polyunsaturated fatty acid content” were enriched for cis- and trans-eQTLs in S2. The traits “total body fat tissue linear” and “loin eye area linear” were enriched for cis-eQTLs in S6 and trans-eQTLs in S2. Additionally, “carcass weight (cold)” was enriched for cis-eQTLs in S2, S4, and S8, and for trans-eQTLs in S6. For the “Production” QTL type, the traits “average daily gain” and “body weight (slaughter)” were enriched for cis- and trans-eQTLs in scenarios S2 and S6 and only for cis-eQTLs in scenarios S4 and S8.

**Fig. 4:**
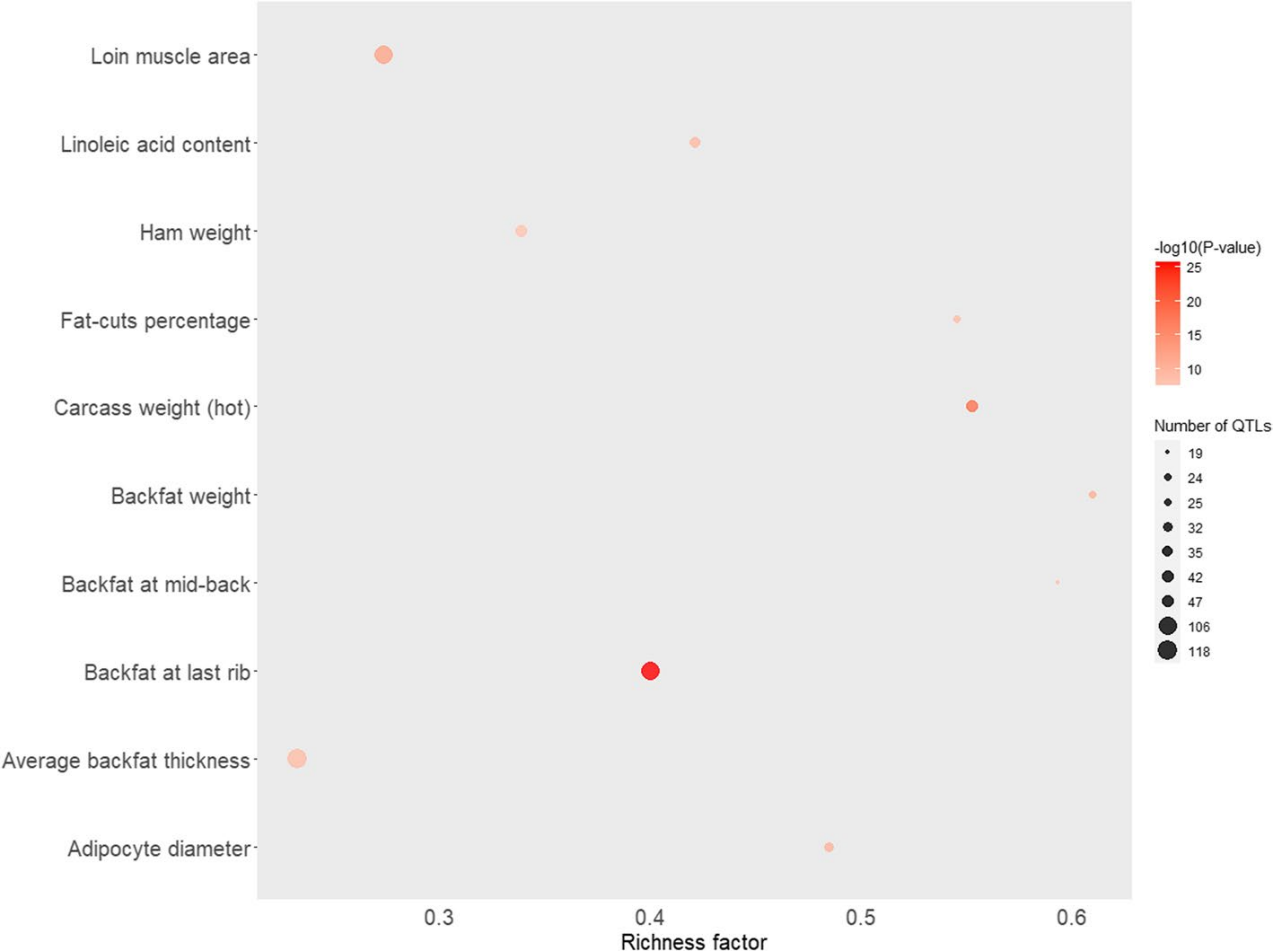
Top 10 significant traits in Meat and Carcass QTL-type enrichment analyzes for cis-eQTLs identified in porcine skeletal muscle. The area of the bubbles represents the number of observed QTL for that class, while the color represents the *p*-value scale (the darker the color, the more significant the *p*-values). Additionally, the X-axis shows the richness factor for each QTL, representing the ratio of the number of QTL and the expected number of that QTL

**Fig. 5:**
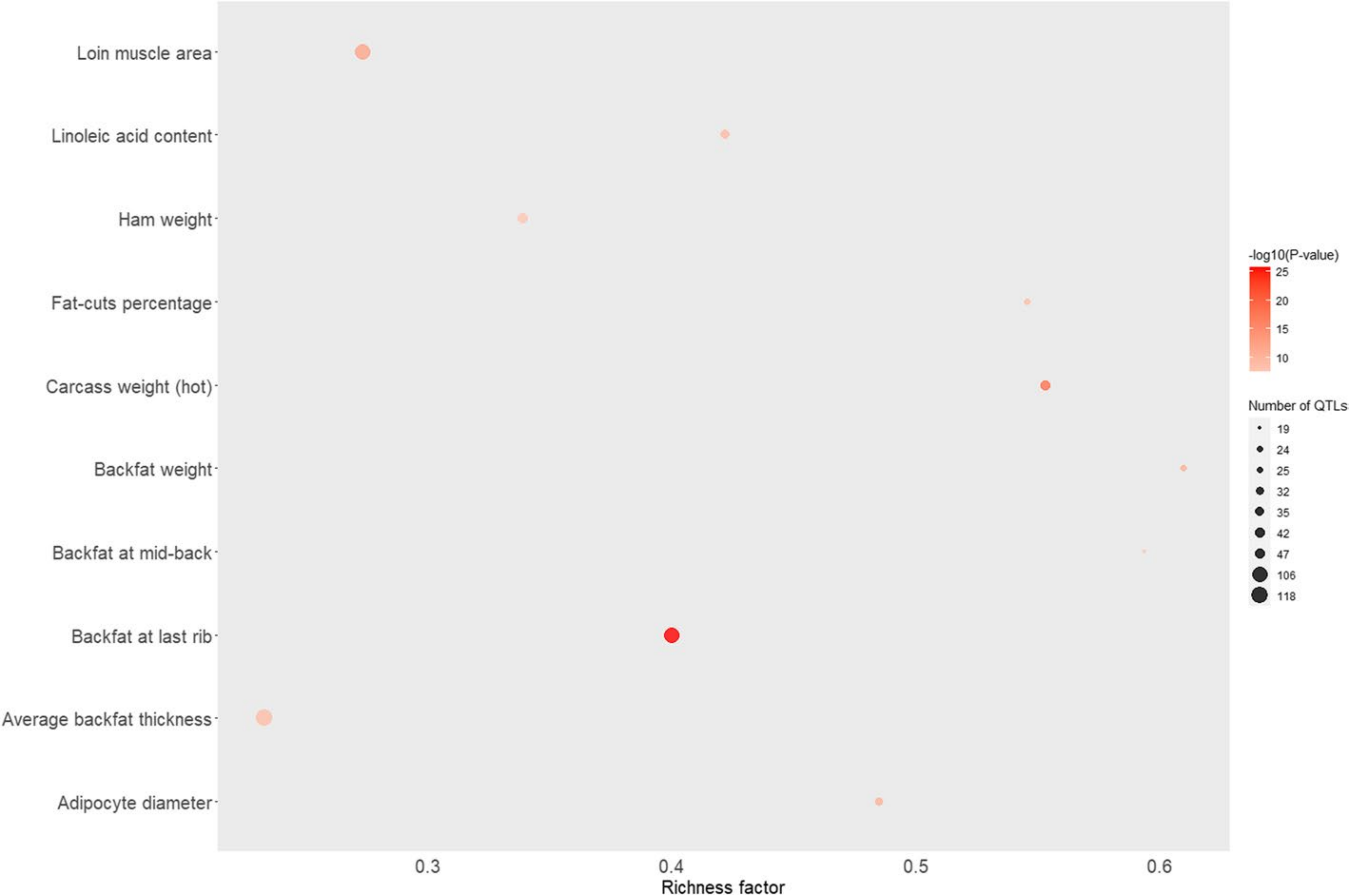
Top 10 significant traits in Meat and Carcass QTL-type enrichment analyzes for trans-eQTLs identified in the porcine skeletal muscle transcriptome. The area of the bubbles represents the number of observed QTL for that class while the colors represent the *p*-value scale (the darker the color, the more significant are the *p*-values). Additionally, the X-axis shows the richness factor for each QTL, representing the ratio of the number of QTLs and the expected number of that QTL

 The QTL type enriched with the SNP markers of the most significant eQTLs was “Meat and Carcass”, followed by “Health” across all scenarios. The top 10 significant traits in the Meat and Carcass QTL type enrichment analyses for cis- and trans-eQTLs from scenario S2 are shown in Figs. 4 and 5. More details about the enrichment results are shown in Additional file 5.

### Gene Ontology (GO), functional annotation, and metabolic pathways

The genes (counted uniquely) regulated by cis- and trans-eQTLs, identified in scenarios S2 (cis = 304, trans = 1,965), S4 (cis = 159, trans = 8), S6 (cis = 185, trans = 5,993), and S8 (cis = 109, trans = 6) were used for Gene Ontology (GO), gene annotation, and metabolic pathway (MP) analyses. The same gene set was used for functional enrichment analyses. These analyzes were performed to understand the biological mechanisms influenced by the candidate genes regulated by cis- and trans-eQTLs.

We first applied a filter on the annotation description of the genes modulated by cis- and trans-eQTLs in each of the scenarios evaluated before investigating eQTLs associated with possible gene regulation mechanisms. We used key terms such as transcription factors, inhibitors, co-regulators, chromatin modelers and remodelers, histone acetylators, modifiers, RNA binding, repressors, and other genes related to gene regulation. Furthermore, we also annotated the genes harboring the identified eQTLs. More details of the gene annotation of the regulated genes found in scenarios S2 to S8 are presented in Additional file 6.

The most significant metabolic pathway in S2, considering the genes regulated in cis-eQTLs type was “Chemical carcinogenesis” (ssc05204). No GO terms were enriched for this gene set. For the genes regulated in trans-eQTLs class in S2, the most enriched GO terms were the biological process (BP) “Small GTPase-mediated signal transduction” (GO:0007264), the molecular function (MF) “calcium ion binding” (GO:0005509), and the cellular component (CC) “cell leading edge” (GO:0031252), and the most significant metabolic pathway was “Adherens junction” (ssc04520). For S4, only two metabolic pathways were enriched, the first and most significant was “Drug metabolism” (ssc00982) considering the genes regulated in cis, with no GO terms enriched for the trans regulated genes. For genes regulated by trans-eQTLs, there was no significant MP, CC, BP, or MF. For S6, four metabolic pathways were enriched and the most significant was “Ovarian steroidogenesis” (ssc04913) in cis action. On the other hand, in trans, the most enriched GO terms for the S6 scenario were the BP “circulatory system development” (GO:0072359), the CC “extrinsic membrane component” (GO:0019898), and the MF “identical protein binding” (GO:0042802), and the MP “Proteoglycans in cancer” (ssc05205). There were no significant GO terms or MP for S8. The most enriched GO terms and MP are shown in Table 4 and further details of the enrichment analyses for the GO domains of BP, MF, CC, and MP are presented in the Additional file 5.

**Table 4:** Description of the most enriched gene ontology (GO) and metabolic pathways (MP) terms across the evaluated scenarios

Gene Set	Scenario	Action	Term	Description	FDR
ssc05204	S2	Cis	MP	Chemical carcinogenesis	4E-05
GO:0007264	S2	Trans	BP	Small GTPase mediated signal transduction	1E-04
GO:0031252			CC	Cell leading edge	1E-04
GO:0005509			MF	Calcium ion binding	4E-05
ssc04520			MP	Adherens junction	2E-08
ssc00982	S4	Cis	MP	Drug metabolism	5E-02
ssc04913	S6	Cis	MP	Ovarian steroidogenesis	4E-04
GO:0072359	S6	Trans	BP	Circulatory system development	3E-11
GO:0019898			CC	Extrinsic component of membrane	1E-04
GO:0042802			MF	Identical protein binding	7E-05
ssc05205			MP	Proteoglycans in cancer	5E-08

*MP *Metabolic pathways, *BP *Biological process, *CC *Cellular component, *MF *Molecular function, *FDR *False Discovery Rate, *S2 *SNPs from the RNA-seq data of the skeletal muscle, *S4 *SNPs from the GGP-50K plus the SNPs from the RNA-seq data of the skeletal muscle, liver, and brain tissues, *S6 *SNPs from the RNA-seq data of the skeletal muscle after linkage disequilibrium pruning (r² threshold of 0.70); cis: genes locally modulated by eQTLs used for the enrichment analyses; trans: genes distantly modulated by eQTLs used for the enrichment analyses

Comparing up to 100 GO terms and the most enriched MP between S2 and S6 trans-eQTLs, there was an overlap of 71.4% of CC terms, 71.4% of MF, and 86.1% of BP. The overlapping results of MP from S2 and S6 revealed 28% and 82.5% of similarity in cis- and trans-eQTLs, respectively. There was a 100% overlap of the significant MP in cis-eQTLs from the S4 and the top twenty most significant pathways in cis-eQTLs for S2.

### Genes modulated by eQTLs

A total of 457 genes were found to be associated with the eQTLs from the different scenarios. The scenarios with SNPs only from the skeletal muscle transcriptome (S2 and S6) enabled the identification of more genes modulated both in cis and in trans action. Scenarios with LD-pruned SNPs also identified more modulated genes. Additionally, the scenario S6 presented the largest number of overlapping genes modulated with the other scenarios, that is, genes modulated in cis or trans identified in scenario S6 were frequently identified in other scenarios.

In S2, the trans-eQTLs located in the genes encoding *CEBZB* (zeta CCAAT enhancer binding protein), *eIF2B* (eukaryotic translation initiation factor 2B subunit alpha), *TSTD3* (sulfur thiosulphate transferase domain containing 3), *TMEM245* (Transmembrane protein 245), and *OXCT1* (3-oxoacid CoA-transferase 1) were identified. These trans-eQTLs were associated with expression of genes involved in regulatory mechanisms, such as transcription factors, chromatin modifiers, primers, and bindings. Key transcription factors identified include *BCLAF1*, *E2F8*, *ELF1*, *ELK3*, *ETS1*, *ETV6*, *GABPB1*, *TCF12*, *TCF4*, *GTF3C1*, *MYT1L*, *SREBF2*, *YY1*, *ETS1*, *SOX7*, *FAP2A*, and *GTF3C5*.

In addition, cis-eQTLs identified in S2 modulate the genes *NAT10* (RNA cytidine acetyltransferase), *YIPF2 (*Protein yipf2 isoform x3; member 2), *EARS2* (Probable glutamate—tRNA ligase, mitochondrial isoform x1; glutamyl-tRNA synthetase 2; Belongs to the class-I aminoacyl-tRNA synthetase family), *GBA* (Glucosylceramidase precursor; *Sus scrofa* glucosidase), *MTERF3* (Transcription termination factor 3, mitochondrial isoform x2), *EMG1* (ENSSSCP00000026081), *SYMPK* (Symplekin isoform x1), and *THYN1* (Thymocyte nuclear protein 1 isoform x1). These genes are modulated by cis-eQTLs predicted to 3’UTR, 5’UTR, downstream, upstream, and missense variants.

The inclusion of SNPs from the 50 K SNP chip array resulted in a lower number of significant modulated genes (FDR < 0.01) between scenarios S2 and S3, as well as between S6 and S7, with a pattern inversely to the increase in the number of SNPs. However, the combination of SNPs from the SNP array with skeletal muscle sequencing SNPs (S3 and S7 scenarios) enabled the identification of 13 specific variants of the GGP-50 K associated with 19 genes, including the Zic family member 5 (*ZIC5*) identified exclusively in the S3 scenario in cis. This gene contains a variant from the GGP-50 K panel (rs81431697). In addition, in the S7 scenario, in trans, there were also genes modulated exclusively by variants derived from the GGP-50 K SNP panel dataset, including *SLC7A1, TRAPPC9, ENSSSCG00000034462, GOLT1A, ENSSSCG00000018018, ENSSSCG00000024765, TTC23*, and *ENSSSCG00000009523*. The other genes identified in scenarios S3 and S7, modulated by SNPs from the GGP-50 K SNP panel dataset, were identified in S6, however, modulated by variants identified in the skeletal muscle transcriptome.

Lastly, in scenarios S4 and S8, there were no significant eQTLs derived from the GGP-50 K SNP panel dataset. The cis-eQTLs, detected only in liver or brain tissue (not identified in the transcriptome of skeletal muscle tissue and GGP-50 K), modulated only four genes in S4 (not identified in S2, S3, S6 and S7), including *CACNG5* (calcium voltage-gated channel auxiliary subunit gamma 5), *IK* (IK cytokine), *RBM46* (RNA binding motif protein 46), and *ZNF821* (zinc finger protein 821), which were cis modulated. Only *IK* and *ZNF821* were identified in S8 and cis modulated.

## Discussion

Currently, most of the GWAS studies for meat quality traits in livestock have used SNPs located primarily in non-coding genomic regions. However, Next Generation Sequencing (NGS) technology has enabled the discovery of thousands of SNPs across the whole transcriptome, which are usually not included in SNP chip arrays. Therefore, in this study we evaluated the impact of combining different sets of SNPs from medium-density SNP chip arrays (i.e., GGP-50 K) and SNPs identified in the transcriptome of pig brain, liver, and skeletal muscle tissues (with and without LD pruning) on the identification of cis- and trans-eQTLs and their association with carcass and body composition traits in Large White pigs. In addition, enrichment analyses were performed using the gene lists identified across the scenarios to reveal GO terms and MP in which these genes are involved. The SNPs were used to identify cis- and trans-eQTLs.

### Identification of cis- and trans-eQTLs in different scenarios

The combination of SNP datasets and LD pruning resulted in eight scenarios that were used to identify cis- and trans-eQTLs. A total of 15,090 genes were identified when considering the gene expression level in the skeletal muscle transcriptome. The number of cis- and trans-eQTLs in all scenarios is within the expected ranges reported in the literature. For instance, Liu et al. [28] detected 10,693 cis-eQTLs and 10,961 trans-eQTLs in the *Longissimus dorsi* muscle of 189 crossbred pigs from Duroc boars crossed with Luchuan sows. Liu et al. [29] reported 3,054 eQTLs, including 1,283 cis-eQTLs and 1,771 trans-eQTLs in skeletal muscle from F2 White Duroc *x* Erhualian pigs. Besides the tissue sampled, there are several other differences among studies, which may explain the variability in the number of cis- and trans-eQTLs identified. These differences include the technique used for measuring gene expression (e.g., RT-qPCR, RNA-seq, and microarray), sequencing coverage depth, breed (e.g., Duroc, Luchuan, Erhualian, Large White, or crossbred animals), sample size, statistical models, covariates used for adjusting the phenotypes, level of correction for population stratification, initial number of SNPs and genes considered (SNP x gene interactions), quality control measures applied to SNPs and genes, method and thresholds used for multiple testing correction, and the significance levels.

In this study, there was a decrease in the number of eQTLs associations in cis- and trans-eQTLs as the number of SNPs increased, likely due to the greater stringency of correction for multiple tests and the reduced sample size, as suggested by Huang et al. [30]. The reduction of significant eQTLs by increasing the weight of the correction method may be due to a greater removal of false positives [30]. Thus, when incorporating SNPs from other genomic regions, such as those identified in the liver and brain tissues, it is important to consider stricter significance thresholds. However, such approaches are necessary to capture variants located in other genomic regions that may contribute to a better understanding of the cellular mechanisms involved in phenotypic variability on the studied traits.

### Inclusion of SNPs from different sources to identify eQTLs

It was found that in S3 and S7, the combination of SNPs from a 50 K SNP chip panel with SNPs from the skeletal muscle transcriptome allowed the detection of additional cis- and trans-eQTLs. The eQTLs identified in scenarios S3 and S7 are specifically derived from SNPs from the GGP-50 K SNP panel dataset. Among these, the *rs81431697* eQTL showed modulation in cis action of the *ZIC5* gene, which is involved in cell differentiation [31].

In scenarios S4 and S8, we also identified local and distant eQTLs that modulate genes not identified in the other scenarios and from SNPs derived from muscle sequencing (S2, S3, S6, and S7). These genes are related to bioprocesses (*CACNG5*) [32], regulation of the immune system and autoimmune disorders (*IK*) [33], and developmental disorders (*RBM46)* [34]. Additionally, the *ZNF821* gene encodes a protein involved in the regulation of the structure and function of DNA (GO:1,990,837) [35]. Thus, verifying the combination of all SNPs allowed the identification of genes, not identified in other scenarios, modulated by eQTLs not identified by the sequencing of skeletal muscle tissue transcriptome. However, the scenarios containing the combinations of all SNPs contributed to the identification of genes cis modulated by eQTLs not present in the transcriptome of the skeletal muscle of pigs. The scenarios S4 and S8 presented the lowest number of genes modulated by trans-eQTLs (8 and 6). Therefore, the approach used in these two scenarios is not indicated for detecting distant effects of variants on gene modulation in the skeletal muscle of pigs.

### Modulated genes by cis- and trans-eQTLs and regulatory mechanisms

Trans-eQTLs identified in the genes *CEBZB, eIF2B, TSTD3, TMEM245*, and *OXCT1*, in scenario S2 modulating gene encoding transcription factors, include *BCLAF1, E2F8, ELF1, ELK3, ETS1, ETV6, GABPB1, TCF12, TCF4, GTF3C1, MYT1L, SREBF2, YY1, ETS1, SOX7, FAP2A*, and *GTF3C5*. This indicates potential indirect regulatory interactions between the genes containing the eQTLs and these transcription factors, by trans modulation. These genes play important roles in the phenotypic expression of traits such as carcass, body composition, and meat quality. *BCLAF1* is involved in the regulation of muscle growth in homologues [36]. *E2F8* is involved in the regulation of cell cycle progression [37], and *ELF1* is involved in the regulation of gene expression [38, 39]. Additionally, chromatin modifiers identified in this study, such as *GABPB1*, *TCF12*, and *GTF3C1*, are known to play a role in regulating gene expression [40–42].

Genes such as *MYT1L*, *SREBF2*, and *YY1* also play important roles in regulating gene expression [43–50], and they may interact with each other, such as enhancer *CEBPZ* and *eIF2B* regulate the expression of genes involved in protein synthesis, potentially impacting skeletal muscle mass. *OXCT1*, on the other hand, can interact with other genes to regulate the expression of genes involved in muscle development [23, 51], potentially affecting meat quality.

Additionally, we highlighted other potential mechanisms such as the cis action –a cis-eQTL modulates the expression of genes nearby. The variants predicted by VEP indicate consequences, such as changes in 3’UTR, downstream gene, upstream gene, and missense regions. These consequences may imply changes in amino acids, molecular affinity, tridimensional structure, or mRNA stability, all of which can affect regulation of gene expression. Changes in the expression of genes such as *TPM1* and *ARL14EP* could influence the regulation of cell growth, and consequently, muscle growth and carcass weight [52]. Genes involved in energy metabolism such as *GLUT4* and *CPT1A* were also identified. For example, *GLUT4* is involved in glucose uptake by cells and *CPT1A* is involved in the production of ketone bodies from fatty acids [46, 53, 54]. Thus, alterations in the expression of these genes can lead to changes in carcass and body composition traits.

### The impact of linkage disequilibrium pruning on eQTL identification

Based on the observed pattern of the scenarios based on LD pruning (S6, S7, and S8), and the fact that more SNPs implies in an increased number of statistical tests and more conservative FDR correction, the number of cis-eQTLs in S8 is the only one that increased across all scenarios compared. In all other cases including trans-eQTLs, the detection sensitivity of eQTLs decreased with the relative increase in the number of SNPs. Although cis-eQTLs are more easily identified [28], they were not the most predominant in this study.

Pruning for LD also had a significant impact on the identification of eQTLs, as genetic variations that are linked may also be associated with differences in gene expression levels. Therefore, LD pruning is important as it allows the removal of linked genetic variants that may confound the results of gene expression analyses [9, 12, 15, 16, 21, 55, 56]. LD pruning reduces the number of variants considered in the analyses, which can improve the results by reducing collinearity among SNPs [15, 21].

The numerical difference in the total association number of cis- and trans-eQTLs identified in S2 was 214, whereas in the equivalent scenario subjected to LD pruning (S6) this difference was 13,430 eQTLs. A similar pattern was observed between scenarios S3 and S7. However, smaller differences were observed when considering only the unique eQTLs. A notable decrease in the count of the cis-eQTLs from S2 (2,247) to S6 (223) was observed, indicating that LD pruning, despite reducing the numerical count of cis-eQTLs and their unique genomic coordinates, favored the identification of trans-eQTLs. The cis effect adopted in these analyses refers to the “local” effect, as explained by Hasin-Brumshtein et at. [57]. These cis-eQTLs are defined by a distance of up to 1 Mb from the regulated gene, indicating that these SNP are closer and thereby more susceptible to be pruned due to greater LD among them. This could explain the reduction in the number of cis-eQTLs from S2 to S6.

It was also observed that most of the genes modulated by eQTLs to GGP-50 K (scenarios S3 and S7) were also modulated by eQTLs from scenario S6. This indicates that LD pruning may contribute to increasing the eQTL detection ability, which would explain part of the overlapping of modulated genes in the scenarios enriched with SNPs from SNP chip arrays such as S3 and S7.

As some of the cis- and trans- eQTLs were associated with several genes, genes associated with several eQTLs simultaneously were also observed. The scenarios with SNPs from skeletal muscle sequencing of pigs identified the greatest number of genes. Additionally, it demonstrated significant overlap in functional analyses with the LD unpruned scenarios, despite having a smaller set of initial SNPs. This suggests that LD pruning can effectively balance the stringency of multiple testing correction. These observations highlight the intrinsic relationship between pruning for LD and FDR in sensibility of the correction to multiple tests from the results.

### eQTL associations with carcass and body composition traits

The cis- and trans-eQTLs identified in each of the scenarios were used for the GWAS analyses with carcass and body composition traits. However, there were no significant variants or any trends for the tested traits. According to Yang et al. [58], MLMA is directly related to the proportion of samples to the number of SNPs and a small number of markers reduces the power of the MLMA model. The lack of significance in our analyses may be related to the small sample size (72 pigs). Larger sample sizes are recommended for future studies [59].

Despite the lack of significant associations between cis- and trans-eQTLs with carcass traits and body composition, a substantial number of overlapping eQTLs with previously reported QTL related to pork meat and carcass traits were identified. This overlap with QTL provides valuable insights into potential regulatory interactions of cis- and trans-eQTLs and gene mechanisms that may influence the carcass and body composition traits evaluated in this study.

The incorporation of SNPs from brain and liver tissues transcriptomes, as well as SNPs from SNP chip arrays, into the skeletal muscle SNP dataset was helpful in identifying genes not identified solely based on SNPs from skeletal muscle transcriptome. However, this increases the number of statistical tests as discussed earlier. LD pruning contributed to increasing the number of eQTLs identified with good concordance with the functional enrichment analyses. However, LD pruning might also lead to the loss of information on potentially important variants. Furthermore, different approaches to combine SNPs can lead to different tested hypotheses. These combinations can increase false positives in addition to inflating errors and increasing the weight of the multiple testing correction. However, the combination of SNP sources and LD pruning depends on the hypothesis to be assessed, in addition to factors such as data availability and sample size. The eQTLs identified in this study can be used in future analyses of gene regulation, cis- and trans-eQTLs-regulated genes, gene co-expression networks, and data integration. However, it is important to note that the sample size used in the study was a limiting factor for some analyses, such as GWAS and associations with the phenotypic traits.

## Conclusions

The scenarios including LD-pruned SNPs (r²>0.7) identified in the transcriptome of the skeletal muscle tissue of pigs resulted in the highest number of genes modulated by eQTLs. The eQTLs identified are involved in gene regulation related to complex traits of pigs, such as transcription factors and enhancers. In addition, combining SNPs identified in the transcriptome of skeletal muscle with SNPs from the transcriptome of brain and liver tissues and SNPs from SNP chip arrays contributed to the identification of eQTL modulating genes not identified when using only the SNPs from the pig skeletal muscle transcriptome. New functional candidate variants associated with the gene expression levels in skeletal muscle were identified in all scenarios. Interestingly, the addition of the 50 K SNP chip array data resulted in gene associations not discovered in the other scenarios. Overall, in this study we identified various novel candidate functional variants associated with the level of gene expression in porcine muscle that contribute to better understanding phenotypic variability in complex traits in pigs.

### Methods

#### Experiment

The transcriptome and the carcass and body composition data used in this study were previously described by our team [25, 60, 61]. Briefly, 72 genetically lean male immunocastrated pigs of the Large White breed with negative genotypes for the homozygous halothane gene (NN) were randomly assigned to one of four dietary treatments with six replicate pens per treatment and three pigs per pen. Treatments consisted of diets supplemented with 1.5% degummed soybean oil or 3% oil from soybean oil, or 3% canola oil, or 3% fish oil from crooked sardines (*Cetengraulis edentulus*). All animals had ad libitum access to feed and water throughout the experimental period (98 days). The average initial body weight (BW) was 28.44 ± 2.95 kg, and the average age was 71 ± 1.8 days. The pigs were fed a basal diet formulated to meet or exceed the nutritional requirements for growing and finishing pigs [62].

### Collection of samples and phenotypes

After a 12-h fasting period, the pigs were slaughtered with an average BW of approximately 132.7 kg. Skeletal muscle (*Longissimus lumborum*) between the 10th and 11th ribs, liver (right lobe of the liver), and brain (portion of the middle region of the frontal lobe) samples were collected within a maximum of 30 min after bleeding, immediately frozen in liquid nitrogen, and then stored at -80° C in an ultra-freezer. These samples were used for total mRNA extraction. The carcass and body compositions phenotypes collected include SW, CCY, LEA, BFT, and IMF [25].

### Total RNA extraction and mRNA sequencing

The RNA extraction from the tissue of skeletal muscle, brain, and liver samples, quality control of the RNA-seq data, counting, and normalization are described in Silva et al. [36] and Fanalli et al. [37, 38]. The sequencing analyses were performed at the Genomics Center from the Luiz de Queiroz College of Agriculture (ESALQ), Piracicaba, São Paulo, Brazil.

### Quality control of RNA-seq data, counting and normalization

The quality of RNA-seq was checked using the FastQC software v. 0.11.8 [39]. Sequencing adapters and low complexity reads were removed by Trim Galore 0.6.5 software [40]. Reads with a minimum length of 70 bases and a *Phred* score greater than 33 were kept after trimming and were aligned and mapped to the porcine reference genome (*Sus scrofa* 11.1) [41] using the assembly available at Ensembl (Release 102) [42]. Alignment, mapping, and sorting (by genomic coordinates) were performed using the STAR v. 2.7.6a software [43].

The dataset used is available in the European Nucleotide Archive (ENA) repository (EMBL-EBI), under the accession PRJEB52665 (brain tissue) [www.ebi.ac.uk/ena/data/view/PRJEB52665]; PRJEB50513 [www.ebi.ac.uk/ena/data/view/PRJEB50513] (liver tissue); and PRJEB52629 (skeletal muscle tissue - *Longissimus lumborum*) [www.ebi.ac.uk/ena/data/view/PRJEB52629].

### Identification of SNPs in RNA-seq data

For the variant calling analyses for each tissue, the Genome Analysis Toolkit (GATK, v. 4.1.9.0) was used in the Genomic Variant Call Format (GVCF) mode [44, 45]. Genome coverage for each of the BAM files was calculated using SAMtools (v. 1.9) [46, 47]. The HaplotypeCaller algorithm [44, 45] was used to call the variants individually, generating GVCF files for each sample. These files were then merged using the CombineGVCF tool [44, 45] and the joint genotyping analyses were performed using the GenotypeGVCF [44, 45]. In the end, a VCF file with all genotypes was generated.

#### DNA extraction and genotyping

The extraction of the genomic DNA of the 72 animals was performed using 30 mg of liver tissue that was macerated in liquid nitrogen, transferred to a 1.5mL microtube, and then processed according to the procedures protocol suggested by the manufacturer of the HighPrep™ Blood & Tissue DNA Plus Kit (MagBio Genomics, London, UK) which uses nucleic acid isolation technology based on magnetic beads. Subsequently, the DNA obtained was evaluated for quality and quantity by readings in the NanoDrop 2000 nano-spectrophotometer (Thermo Fisher Scientific, Waltham, MA, USA) at three different wavelengths 230 nm, 260 nm, and 280 nm. The integrity of the DNA extracted from the samples was evaluated by means of Ultra-Violet (UV) light visualization of the electrophoresis run on a 1.5% agarose gel [w/vol] and in Tris-borate-EDTA buffer with GelRed fluorescent staining (Biotium, Hayward, CA, USA). After DNA evaluation and quantification, an aliquot of approximately 1,000 ng was sent to the NEOGEN company (Pindamonhangaba, SP, Brazil) for genotyping using the first generation GeneSeek Genomic Profiler (GGP) Porcine 50 K, a medium-density SNP chip array with 50,915 SNPs. The results were received in the Illumina raw format and converted to PLINK 1.9 [48] “ped” and “map” formats using a python algorithm [https://github.com/bioinformatics-ptp/Zanardi/blob/master/Zanardi.py]. The individual identification (iid) and family identification (fid) referring to the animals were updated by PLINK 1.9 [48], based on the sampling index present in the “sample map” file, so that the animal identifications coincided with the other data files. In addition, the genomic coordinates (position and chromosome) were updated for the latest version of the Illumina GGP Porcine 50 K-24 v2 chip (www.illumina.com/products/by-type/microarray-kits/ggp-porcine.html). Data from SNPs from all tissues and 50 K animal genotyping were merged (--bmerge) using the PLINK 1.9 software [48].

### Quality control

After the variant calling, to reduce the false discovery rate, variants were filtered by the SNP for variant quality score (QUAL) equal or greater than 30 (*Pred* score, Sanger/Illumina 1.9 + encoding) [46, 49, 50] and total coverage depth (DP) equal or greater than 10, using BCFtools v. 1.9. [24, 46]. Subsequently, we filtered only SNPs of the autosomal chromosomes from 1 to 18 and biallelic SNPs using the PLINK 1.9 software [23, 48]. Quality filters were used for variants with low MAF (--maf) 0.05, variants with a 0.95 genotyping call rate (0.05 missing) (--geno), and variants with extreme departure from the Hardy-Weinberg equilibrium test (--hwe) with *p*-value lower than 10^−6^ [23, 24, 51, 52]. Files were also generated without quality filters to perform LD pruning considering a r² threshold of 0.70 (--indep-pairphase) in the PLINK 1.9 software [63]. The parameters used for LD pruning were: “--indep-pairphase 50 5 0.7”, that is, a window size equal to 50 SNPs, a window offset every 5 SNPs per step, and a correlation threshold (r²) paired equal to 70%. Thus, the pairs of SNPs in each window of 50 SNPs, with a square correlation greater than 70% were noted and one of the SNPs of that pair was removed, later, the window was shifted by 5 SNPs, and the procedure was repeated, until none of these correlated pairs (r²>0.7) remained [48]. The genomic datasets pruned for LD were subsequently filtered for MAF, missing call rate, and extreme departure from HWE as previously described.

#### Scenarios

To identify the SNPs that modulate the level of gene expression in the skeletal muscle of pigs, the SNPs identified from the brain and liver transcriptome data and 50 K genotyping were combined with the skeletal muscle transcriptome SNP dataset, which generated four datasets (scenarios). These datasets were subjected to LD pruning, which resulted in four additional scenarios. These combinations were performed to investigate the impact of adding SNPs from different tissues and SNP identification methods to SNPs derived from skeletal muscle transcriptome sequencing data on the identification of cis- and trans-eQTLs in muscle. Furthermore, LD pruning was performed for all combinations of SNPs, aiming to elucidate the implications of using this technique in the eQTLs mapping, according to different combinations of SNPs from different tissues and methods for SNP identification. These scenarios are: (S1) SNP set from the GGP-50 K; (S2) SNP set from the SNP calling of RNA-seq data of the skeletal muscle; (S3) SNP set from the GGP-50 K plus the SNP calling of RNA-seq data of the skeletal muscle; (S4) SNP set from the GGP-50 K plus the SNP calling of RNA-seq data of the skeletal muscle, liver, and brain tissues; (S5) SNP set from the GGP-50 K after LD pruning; (S6) SNP set from the SNP calling of RNA-seq data of the skeletal muscle after LD pruning; (S7) SNP set from the GGP-50 K plus the SNP calling of RNA-seq data of the skeletal muscle after LD pruning; and, (S8) SNP set from the GGP-50 K plus the SNP calling of RNA-seq data of the skeletal muscle, liver, and brain tissues after LD pruning.

#### Identification of eQTLs

The Matrix eQTL package [53] of the R statistical program was used to identify associations between the SNPs from different scenarios and the gene expression level of the skeletal muscle tissue. The window for cis-eQTL (local effect) was defined as up to 1 Mb upstream from the start of transcription and up to 1 Mb downstream from the end of the regulated gene. The other combinations were considered as trans-eQTL. To deal with gene expression outliers, the data was transformed to a normal distribution based on the mean, preserving the relative rank, as recommended by the consortium ‘GTEx’ (Genotype-Tissue Expression) [54]. The Matrix eQTL package tests the linear association between each marker (SNP) and gene assuming the genotype effect as additive, performs a separate test for each pair (marker and gene), and corrects for multiple testing by calculating the false discovery rate (FDR) [60, 61]. The fixed linear regression model fitted was:

$\mathrm G\;=\;\mathrm\beta\ast\mathrm s\;+\;\mathrm{PC}\;+\;\mathrm{SBW}\;+\;\mathrm{SIRE}\;+\;\mathrm{THREAT}\;+\;\mathrm\varepsilon$ where $\text{G}$ is the gene expression level in normalized transcripts per million (TPM), ${\upbeta }$ is the SNP allelic substitution effect, $s$ is the genetic marker covariate, coded as 0 (homozygous for the reference allele), 1 (heterozygous), and 2 (homozygous for the reference alternative), $\text{PC}$are the first 10 principal components to correct for potential population stratification (these principal components explained a total of 28% on the variance-standardized relationship matrix), $\text{SBW}$ is the initial body weight, $\text{SIRE}$ is a dummy variable that represents the sire effect, TREAT is a dummy variable that represents the treatment effect, and $\varepsilon$ is the random residuals with $\varepsilon\sim\text{i.i.d.N}\left(0,\sigma^2\right)$. For both cis- and trans-eQTLs, an FDR level of 0.01 was considered.

The estimated effect size (slope coefficient) and the genetic variance explained by the markers were also calculated according to the Matrix eQTL package [53]. The scatter plots were done using the R ggplot2 package [64]. The genomic coordinates of the eQTLs data and associated genes were converted to mega base pairs (Mb) and sorted by chromosome and position. The eQTLs and gene position orders were used to plot the graphs with the X-axis referring to the order of the SNPs and the Y-axis referring to the order of the initial position of the genes.

#### Association with carcass and body composition traits

After identifying the eQTLs, significant SNPs were selected and these SNPs were associated with the traits of interest. The association between the SNPs in the eQTLs for all scenarios (S2, S3, S4, S6, S7, and S8) with the phenotypes was performed using a Mixed Linear Model Association (MLMA) [57] in the GCTA software [65], considering the effects identified for each phenotype and fitting the genomic relationship matrix to account for population stratification and polygenic effects. The model fitted was:

$\text{y}=\text{a}+\beta\text{*x}+\text{SBW}+\text{TREAT}+\text{G}+\varepsilon$ where $\text{y}$ is the phenotypic record of each carcass and body composition trait evaluated, $\text{a}$ is the overall mean, ${\upbeta }$ is the additive effect (fixed effect) of the SNP being tested for potential association with the phenotype, $\text{x}$ is the indicator variable of the SNP genotype coded as 0 (homozygous for the reference allele), 1 (heterozygous), or 2 (homozygous for the reference alternative), SBW is the systematic effect of initial body weight (as a linear covariate), TREAT represents the systematic treatments effect, $\text{g}$ is the polygenic effect (random effect), that is, the cumulative effect of all SNPs (as modelled by the genomic relationship matrix), and $\varepsilon$ is the residual effect. The carcass and body composition traits evaluated were SW, CCY, LEA, BFT, and IMF.

The values resulting from the association analyses were corrected for multiple tests using the FDR method [30, 60], and the significance level adopted was 5% while we considered as indicative the FDR value between 5% and 10%.

### Prediction of the effects of cis- and trans-eQTLs identified in each of the scenarios

Based on the significant cis- and trans-eQTLs variants (FDR < 0.01) identified in scenarios S2, S4, S6, and S8, the functional consequence analyses of these variants (cis plus trans) for each of these scenarios were predicted using the VEP tool (Ensembl release 109 - Feb 2023 © EMBL-EBI) [66] and considering the *Sus scrofa* 11.1assembly genome. The distance to transcription that the VEP assigned for the upstream/downstream consequence was of up to 5,000 bp and the other configurations were kept the default of the web interface (www.ensembl.org/info/docs/tools/vep/online/index.html) [67]. The command line used was “./vep --appris --biotype --buffer_size 5000 --check_existing --distance 5000 --mane --sift b --species sus_scrofa --symbol --transcript_version --tsl --cache --input_file [input_data] --output_file [output_file]”, where input_data is the VCF file with the cis- and trans-eQTLs from each scenario.

### Annotation and functional enrichment of eQTLs

The GALLO package [26] was used to perform the QTL annotation of the SNPs identified as cis- and trans-eQTLs in scenarios S2, S4, S6, and S8. The eQTLs annotation was performed using known QTL data obtained from the PigQTLdb database (version 47 - pigSS11) [27], considering a window of up to 100 kb downstream and upstream of the genomic coordinates of the cis- and trans-eQTLs for each scenario. Enrichment analyses were performed using a hypergeometric test based on the “qtl_enrich” function from the GALLO package [26] to reduce the bias of overrepresented traits. The QTL enrichment test was performed using traits annotated within the candidate regions (window of up to ± 100 kb of the eQTL) from the QTL database, considering 18 autosomes. The hypergeometric test estimate allows us to determine whether the number of records observed for a specific trait in the 18 pig autosomes is greater than what would be expected by chance.

## Gene annotation, GO, and metabolic pathways for the genes

Annotations of genes close to the cis- and trans-eQTLs were performed in each of the scenarios (S2, S4, S6, and S8), considering a window of 100 kb downstream and upstream of each eQTL. The adopted reference position was the genomic coordinate of each of the cis- and trans-eQTL, for each scenario. Data from the gene annotation of the species *Sus scrofa* (Assembly *Sscrofa*11.1; genome-build-accession GCA_000003025.6; available at: [https://ftp.ensembl.org/pub/release-106/gtf/sus_scrofa/]) were extracted from the Ensembl platform (Ensembl release 106 - Jul 2022) [42] in the “.gtf” format (gene transfer format).

Only genes regulated by cis- and trans-eQTLs (FDR < 0.01) were selected for the GO and MP analyses to understand the functional roles of the genes related to the eQTLs. These analyzes were performed using the WebGestaltR package [68], where counted genes uniquely modulated by cis- and trans-eQTLs in each scenario (S2, S4, S6, S8) were used to identify biological processes (BP), molecular functions (MF), cellular components (CC), and metabolic pathways (MP). The enrichment method adopted was ORA (Over-Representation Analyses), while the other settings were the package default for each set of genes regulated by cis- and trans-eQTLs, for each scenario separately.

### Supplementary Information

** Additional file 1.** “Composition of scenarios by the method of identification of SNPs by along the scenarios”. Description: This file presents the count of the SNPs by their respective methods of obtained dataset, RNA-seq and GGP-k. This count was made after quality control filters and could be used to understand the composition of scenarios.

** Additional file 2.** “Cis- and trans-eQTLs identified based on different scenarios of genomic data from pigs”. Description: This file presents the results of the expression quantitative trait loci (eQTLs) analyses for different scenarios (FDR < 0.01). It includes information on the eQTLs identified in each scenario, providing insights into the genetic variants associated with gene expression regulation. Furthermore, the information of the VEP is included to facilitate to understand the information about the cis- and trans-eQTLs.

** Additional file 3.** “Manhattan plots and QQ-plots for respective scenarios (S1-S8)”. Description: This file shows all Manhattan plots from GWAS analyses above the scenarios S2, S3, S4, S6, S7, and S8.

** Additional file 4.** “Variant Effect Prediction for cis- and trans-eQTLs identified in the skeletal muscle of pigs with threshold of 1% from the FDR”. Description: This file provides Variant Effect Prediction (VEP) information for all cis- and trans-eQTL (FDR < 0.01) identified in the scenarios S2, S3, S4, S6, S7, and S8. It includes detailed annotations and predictions on the functional consequences of genetic variants associated with gene expression regulation in the scenarios (FDR < 0.01).

** Additional file 5.** “Annotation of the genes regulated by the cis- and trans-eQTLs based on different scenarios”. Description: This file contains the annotation of the genes regulated by the eQTLs found in scenarios S2, S3, S4, S6, S7, and S8. It provides information on the biological functions, description, and other relevant annotations for the identified genes.

** Additional file 6.** “Annotation and enrichment for the modulated genes by eQTLs identified in the skeletal muscle of pigs”. Description: This file contains the results of gene enrichment analyses, including enriched gene sets, pathways, and functional categories associated with the eQTLs and their regulated genes. This file also provides modulated gene annotations, including gene symbols, chromosomal locations, gene descriptions, and other relevant information.

## Data Availability

The dataset (s) supporting the conclusions of this article is (are) included within the article and its Additional files 1, 2, 3, 4, 5 and 6. The dataset used is available in the European Nucleotide Archive (ENA) repository (EMBL-EBI), under accession PRJEB52665 (brain tissue) [www.ebi.ac.uk/ena/data/view/PRJEB52665]; PRJEB50513 [www.ebi.ac.uk/ena/data/view/PRJEB50513] (liver); and PRJEB52629 (skeletal muscle) [www.ebi.ac.uk/ena/data/view/PRJEB52629].

## Abbreviations

3’UTR: 3 prime untranslated region variant
5’UTR: 5 prime untranslated region variant
BFT: Backfat thickness measured by ultrasound in cm
BP: Biological Process
CC: Cellular Component
CCY: Cold carcass yield in percentage of the slaughter weight
CV: Coefficient of variation
DNA: Deoxyribonucleic acid
DP: Total coverage depth
EMBL-EBI: European Molecular Biology Laboratory-European Bioinformatics Institute
ENA: European Nucleotide Archive
eGWAS: Expression Genome-Wide Association Study
eQTLs: Expression Quantitative Trait Loci
eQTL: Expression Quantitative Trait Locus
FDR: False Discovery Rate
GGP-50K: GeneSeek Genome Porcine medium density SNPs from SNP array
GO: Gene Ontology
GRM: Genomic Relationship Matrix between the pair of animals
GVCF: Genomic Variant Calling Format
HWE: Hardy-Weinberg Exact balance test
Kb: Kilobase (1,000 base pairs)
LD: Linkage disequilibrium
LEA: Loin eye area measured by ultrasound in cm^2^
MAF: Minor allele frequency
Mb: Mega base pair
MF: Molecular function
IMF: Muscle fat content in percentage
MLMA: Mixed Linear Model Association
MP: Metabolic pathways
N: Number
ORA: Over Representation Analyses
PC: Principal components
QTL: Quantitative trait loci
QUAL: *Phred* score
RNA: Ribonucleic acid
RNA-seq: RNA sequencing
RT-PCR: Real Time Polymerase Chain Reaction
RT-qPCR: Real Time Quantitative Polymerase Chain Reaction
r^2^: Correlation
SBW: Initial body weight
SD: Phenotypic standard deviation
SM: Skeletal muscle
SNP: Single nucleotide polymorphism
SSC1: *Sus scrofa* chromosome 1
SSC18: *Sus scrofa* chromosome 18
SW: Slaughter weight in kg
TPM: Transcripts per million
UV: Ultra-violet light
VEP: Variant Effect Predictor
VCF: Variant calling format

## References

[1] Delpuech E, Aliakbari A, Labrune Y, Fève K, Billon Y, Gilbert H (2021). Identification of genomic regions affecting production traits in pigs divergently selected for feed efficiency. Genet Selection Evol.

[2] Ellen E, van der Sluis M, Siegford J, Guzhva O, Toscano M, Bennewitz J (2019). Review of Sensor technologies in animal breeding: phenotyping behaviors of laying hens to select against feather pecking. Animals.

[3] Ramayo-Caldas Y, Mármol-Sánchez E, Ballester M, Sánchez JP, González-Prendes R, Amills M (2019). Integrating genome-wide co-association and gene expression to identify putative regulators and predictors of feed efficiency in pigs. Genet Selection Evol.

[4] Dai Q, Zhou G, Zhao H, Võsa U, Franke L, Battle A (2023). OTTERS: a powerful TWAS framework leveraging summary-level reference data. Nat Commun.

[5] Li B, Ritchie MD (2021). From GWAS to Gene: Transcriptome-Wide Association Studies and other methods to functionally understand GWAS discoveries. Front Genet.

[6] Mancuso N, Shi H, Goddard P, Kichaev G, Gusev A, Pasaniuc B (2017). Integrating gene expression with summary association statistics to identify genes associated with 30 complex traits. Am J Hum Genet.

[7] Moqa R, Younas I, Bashir M (2022). Assessing effectiveness of many-objective evolutionary algorithms for selection of tag SNPs. PLoS ONE.

[8] Zhao Y, Wang K, Wang WL, Yin TT, Dong WQ, Xu CJ (2019). A high-throughput SNP discovery strategy for RNA-seq data. BMC Genomics.

[9] Grossi DA, Jafarikia M, Brito LF, Buzanskas ME, Sargolzaei M, Schenkel FS (2017). Genetic diversity, extent of linkage disequilibrium and persistence of gametic phase in Canadian pigs. BMC Genet.

[10] Daetwyler HD, Kemper KE, van der Werf JHJ, Hayes BJ (2012). Components of the accuracy of genomic prediction in a multi-breed sheep population. J Anim Sci.

[11] De Roos APW, Hayes BJ, Spelman RJ, Goddard ME (2008). Linkage disequilibrium and persistence of phase in Holstein-Friesian, Jersey and Angus cattle. Genetics.

[12] Zhang Y, Wan Q, Cheng X, Lu G, Wang S, He S (2022). A tagging SNP set Method based on Network Community Partition of Linkage Disequilibrium and node centrality. Curr Bioinform.

[13] Wang S, Liu G, Wang X, Zhang Y, He S, Zhang Y (2021). TagSNP-set selection for genotyping using integrated data. Futur Gener Comput Syst.

[14] Arcos-Burgos M, Muenke M (2002). Genetics of population isolates. Clin Genet.

[15] Slatkin M (1994). Linkage disequilibrium in growing and stable populations. Genetics.

[16] Steibel JP, Bates RO, Rosa GJM, Tempelman RJ, Rilington VD, Ragavendran A (2011). Genome-wide linkage analysis of global gene expression in Loin muscle tissue identifies candidate genes in pigs. PLoS ONE.

[17] Carlson CS, Eberle MA, Rieder MJ, Yi Q, Kruglyak L, Nickerson DA (2004). Selecting a maximally informative set of single-nucleotide polymorphisms for Association analyses using linkage disequilibrium. Am J Hum Genet.

[18] Nyholt DR (2004). A simple correction for multiple testing for single-nucleotide polymorphisms in linkage disequilibrium with each other. Am J Hum Genet.

[19] Polizel GHG, Cesar ASM, Cracco RC, Fernandes AC, Reginato GM, Xavier PLP (2022). Identification of eQTLs and differential gene expression associated with fetal programming in beef cattle. J Appl Genet.

[20] Brown CD, Mangravite LM, Engelhardt BE (2013). Integrative modeling of eQTLs and Cis-Regulatory Elements suggests mechanisms underlying cell type specificity of eQTLs. PLoS Genet.

[21] VanLiere JM, Rosenberg NA (2008). Mathematical properties of the r^2^ measure of linkage disequilibrium. Theor Popul Biol.

[22] Service S (2006). Magnitude and distribution of linkage disequilibrium in population isolates and implications for genome-wide association studies. Nat Genet.

[23] Wigginton JE, Cutler DJ, Abecasis GR (2005). A note on exact tests of hardy-Weinberg Equilibrium. Am J Hum Genet.

[24] Li H (2011). A statistical framework for SNP calling, mutation discovery, association mapping and population genetical parameter estimation from sequencing data. Bioinformatics.

[25] Almeida VV, Silva JPM, Schinckel AP, Meira AN, Moreira GCM, Gomes JD (2021). Effects of increasing dietary oil inclusion from different sources on growth performance, carcass and meat quality traits, and fatty acid profile in genetically lean immunocastrated male pigs. Livest Sci.

[26] Fonseca PAS, Suárez-Vega A, Marras G, Cánovas Á (2020). GALLO: an R package for genomic annotation and integration of multiple data sources in livestock for positional candidate loci. Gigascience..

[27] Hu Z-L, Park CA, Reecy JM (2022). Bringing the animal QTLdb and CorrDB into the future: meeting new challenges and providing updated services. Nucleic Acids Res.

[28] Liu Y, Liu X, Zheng Z, Ma T, Liu Y, Long H (2020). Genome-wide analysis of expression QTL (eQTL) and allele-specific expression (ASE) in pig muscle identifies candidate genes for meat quality traits. Genet Selection Evol.

[29] Liu Y, Long H, Feng S, Ma T, Wang M, Niu L (2021). Trait correlated expression combined with eQTL and ASE analyses identified novel candidate genes affecting intramuscular fat. BMC Genomics.

[30] Huang QQ, Ritchie SC, Brozynska M, Inouye M (2018). Power, false discovery rate and winner’s curse in eQTL studies. Nucleic Acids Res.

[31] He X, Tan C, Li Z, Zhao C, Shi J, Zhou R (2019). Characterization and comparative analyses of transcriptomes of cloned and in vivo fertilized porcine pre-implantation embryos. Biol Open.

[32] Kim I-S, Yang S-Y, Han J-H, Jung S-H, Park H-S, Myung C-S (2015). Differential Gene expression in GPR40-Overexpressing pancreatic β-cells treated with linoleic acid. Korean J Physiol Pharmacol.

[33] Iwanami N, Higuchi T, Sasano Y, Fujiwara T, Hoa VQ, Okada M (2008). WDR55 is a nucleolar modulator of ribosomal RNA synthesis, cell cycle progression, and Teleost Organ Development. PLoS Genet.

[34] Qian B, Li Y, Yan R, Han S, Bu Z, Gong J (2022). RNA binding protein RBM46 regulates mitotic-to-meiotic transition in spermatogenesis. Sci Adv.

[35] Nonaka Y, Muto H, Aizawa T, Okabe E, Myoba S, Yokoyama T (2010). STPR, a 23-Amino acid Tandem repeat domain, found in the human function-unknown protein ZNF821. Biochemistry.

[36] da Silva BP, Fanalli SL, Gomes JD, de Almeida VV, Fukumasu H, Freitas FAO (2023). Brain fatty acid and transcriptome profiles of pig fed diets with different levels of soybean oil. BMC Genomics.

[37] Fanalli SL, da Silva BPM, Gomes JD, Ciconello FN, de Almeida VV, Freitas FAO (2022). Effect of dietary soybean oil inclusion on liver-related transcription factors in a pig model for metabolic Diseases. Sci Rep.

[38] Fanalli SL, da Silva BPM, Gomes JD, Durval MC, de Almeida VV, Moreira GCM (2023). RNA-seq transcriptome profiling of pigs’ liver in response to diet with different sources of fatty acids. Front Genet.

[39] Brown J, Pirrung M, McCue LA (2017). FQC Dashboard: integrates FastQC results into a web-based, interactive, and extensible FASTQ quality control tool. Bioinformatics.

[40] Krueger F, James F, Ewels P, Afyounian E, Weinstein M, Schuster-Boeckler B et al. FelixKrueger/TrimGalore: v0.6.10 - add default decompression path. 2023. 10.5281/ZENODO.7598955.

[41] Warr A, Affara N, Aken B, Beiki H, Bickhart DM, Billis K (2020). An improved pig reference genome sequence to enable pig genetics and genomics research. Gigascience.

[42] Aken BL, Ayling S, Barrell D, Clarke L, Curwen V, Fairley S (2016). The Ensembl gene annotation system. Database (Oxford).

[43] Dobin A, Davis CA, Schlesinger F, Drenkow J, Zaleski C, Jha S (2013). STAR: Ultrafast universal RNA-seq aligner. Bioinformatics.

[44] Franke KR, Crowgey EL (2020). Accelerating next generation sequencing data analysis: an evaluation of optimized best practices for genome analysis Toolkit algorithms. Genomics Inf.

[45] Van der Auwera GA, Carneiro MO, Hartl C, Poplin R, del Angel G, Levy-Moonshine A (2013). From FastQ Data to High-Confidence Variant Calls: The Genome Analysis Toolkit Best Practices Pipeline. Curr Protoc Bioinformatics.

[46] Danecek P, Bonfield JK, Liddle J, Marshall J, Ohan V, Pollard MO (2021). Twelve years of SAMtools and BCFtools. Gigascience..

[47] Li H, Handsaker B, Wysoker A, Fennell T, Ruan J, Homer N (2009). The sequence Alignment/Map format and SAMtools. Bioinformatics.

[48] Chang CC, Chow CC, Tellier LC, Vattikuti S, Purcell SM, Lee JJ (2015). Second-generation PLINK: rising to the challenge of larger and richer datasets. Gigascience.

[49] Ewing B, Hillier L, Wendl MC, Green P (1998). Base-calling of automated sequencer traces using Phred. I. Accuracy assessment. Genome Res.

[50] Ewing B, Green P (1998). Base-calling of automated sequencer traces using Phred. II. Error probabilities. Genome Res.

[51] Altmann A, Weber P, Bader D, Preuß M, Binder EB, Müller-Myhsok B (2012). A beginners guide to SNP calling from high-throughput DNA-sequencing data. Hum Genet.

[52] Nielsen R, Paul JS, Albrechtsen A, Song YS (2011). Genotype and SNP calling from next-generation sequencing data. Nat Rev Genet.

[53] Shabalin AA (2012). Matrix eQTL: ultra-fast eQTL analysis via large matrix operations. Bioinformatics.

[54] Aguet F, Anand S, Ardlie KG, Gabriel S, Getz GA, Graubert A (1979). The GTEx Consortium atlas of genetic regulatory effects across human tissues. Science.

[55] Amaral AJ, Megens H-J, Crooijmans RPMA, Heuven HCM, Groenen MAM (2008). Linkage disequilibrium decay and haplotype block structure in the Pig. Genetics.

[56] Weir BS (1979). Inferences about linkage disequilibrium. Biometrics.

[57] Hasin-Brumshtein Y, Hormozdiari F, Martin L, van Nas A, Eskin E, Lusis AJ (2014). Allele-specific expression and eQTL analysis in mouse adipose tissue. BMC Genomics.

[58] Yang J, Zaitlen NA, Goddard ME, Visscher PM, Price AL (2014). Advantages and pitfalls in the application of mixed-model association methods. Nat Genet.

[59] Loh P-R, Tucker G, Bulik-Sullivan BK, Vilhjálmsson BJ, Finucane HK, Salem RM (2015). Efficient bayesian mixed-model analysis increases association power in large cohorts. Nat Genet.

[60] Benjamini Y, Hochberg Y (1995). Controlling the false Discovery rate: a practical and powerful Approach to multiple testing. J Roy Stat Soc: Ser B (Methodol).

[61] Raven LA, Cocks BG, Kemper KE, Chamberlain AJ, Vander Jagt CJ, Goddard ME (2016). Targeted imputation of sequence variants and gene expression profiling identifies twelve candidate genes associated with lactation volume, composition and calving interval in dairy cattle. Mamm Genome.

[62] Rostagno HS, Albino LFT, Donzele JL, Gomes PC, de Oliveira RF, Lopes DC (2011). Tabelas brasileiras para aves e suínos. Composição De Alimentos E exigências Nutricionais.

[63] Purcell S, Neale B, Todd-Brown K, Thomas L, Ferreira MA, Bender D, Maller J, Sklar P, de Bakker PI, Daly MJ, et al. PLINK: a tool set for whole-genome association and population-based linkage analyses. Am J Hum Genet. 2007;81(3):559–75. 10.1086/519795 PMC1950838 17701901

[64] Wickham H. ggplot2. New York, NY: Springer New York; 2009.

[65] Yang J, Lee SH, Goddard ME, Visscher PM (2011). GCTA: A Tool for Genome-wide Complex Trait Analysis. Am J Hum Genet.

[66] Cunningham F, Allen JE, Allen J, Alvarez-Jarreta J, Amode MR, Armean IM (2022). Ensembl 2022. Nucleic Acids Res.

[67] McLaren W, Gil L, Hunt SE, Riat HS, Ritchie GRS, Thormann A (2016). The Ensembl variant effect predictor. Genome Biol.

[68] Liao Y, Wang J, Jaehnig EJ, Shi Z, Zhang B (2019). WebGestalt 2019: gene set analysis toolkit with revamped UIs and APIs. Nucleic Acids Res.

[69] Vaughn SE (2012). Review of the Third Edition of the guide for the Care and Use of Agricultural animals in Research and Teaching. J Am Assoc Lab Anim Sci.

