# Supplementary material for: Identification of eQTLs using different sets of single nucleotide polymorphisms associated with carcass and body composition traits in pigs
Source: BMC Genomics. 2024 Jan 2;25:14. doi: 10.1186/s12864-023-09863-8 (PMC10759680; doi:10.1186/s12864-023-09863-8)
Supplement: Supplementary file 1 — Additional file 1. “Composition of scenarios by the method of identification of SNPs by along the scenarios”. Description: This file presents the count of the SNPs by their respective methods of obtained dataset, RNA-seq and GGP-k. This count was made after quality control filters and could be used to understand the composition of scenarios. [file 12864_2023_9863_MOESM1_ESM.docx]

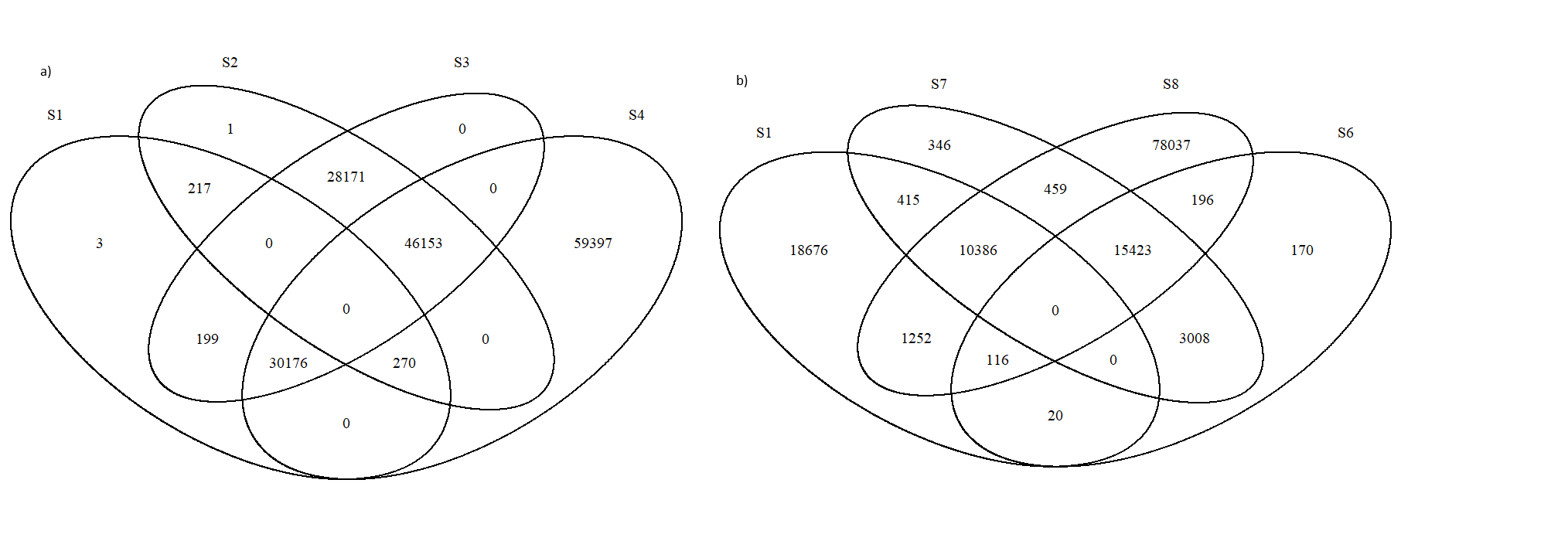


Additional Figure 1. Venn plot diagram of the SNP composition along the scenarios. a. The relationship between scenarios S1, S2, S3 and S4. b. The relationship between scenarios S1, S6, S7 and S8.
