# Supplementary material for: Identification of eQTLs using different sets of single nucleotide polymorphisms associated with carcass and body composition traits in pigs
Source: BMC Genomics. 2024 Jan 2;25:14. doi: 10.1186/s12864-023-09863-8 (PMC10759680; doi:10.1186/s12864-023-09863-8)
Supplement: Supplementary file 3 — Additional file 3. “Manhattan plots and QQ-plots for respective scenarios (S1-S8)”. Description: This file shows all Manhattan plots from GWAS analyses above the scenarios S2, S3, S4, S6, S7, and S8. [file 12864_2023_9863_MOESM3_ESM.pptx]

## Slide 1
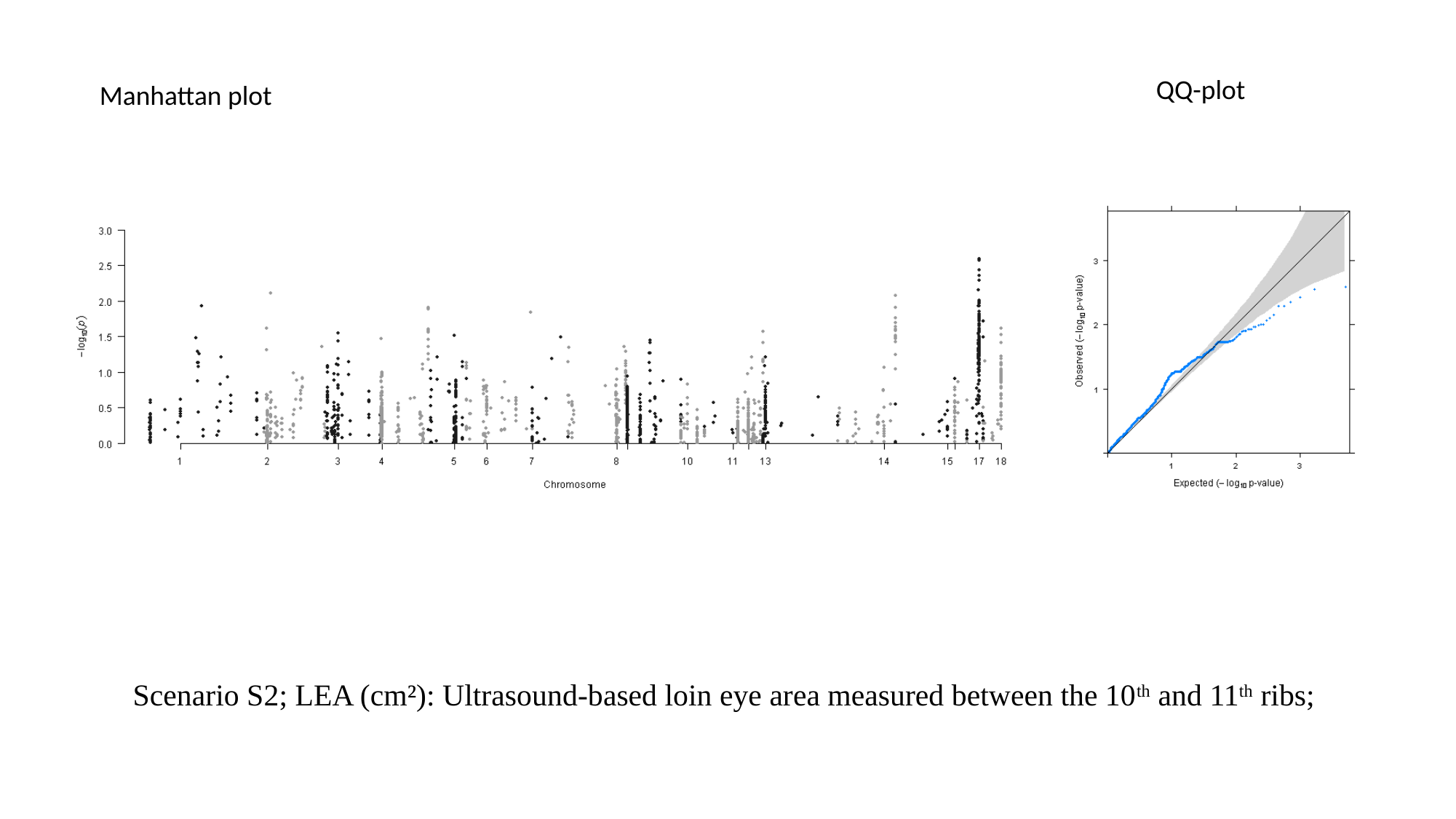

QQ-plot
Manhattan plot
# Scenario S2; LEA (cm²): Ultrasound-based loin eye area measured between the 10th and 11th ribs;

## Slide 2
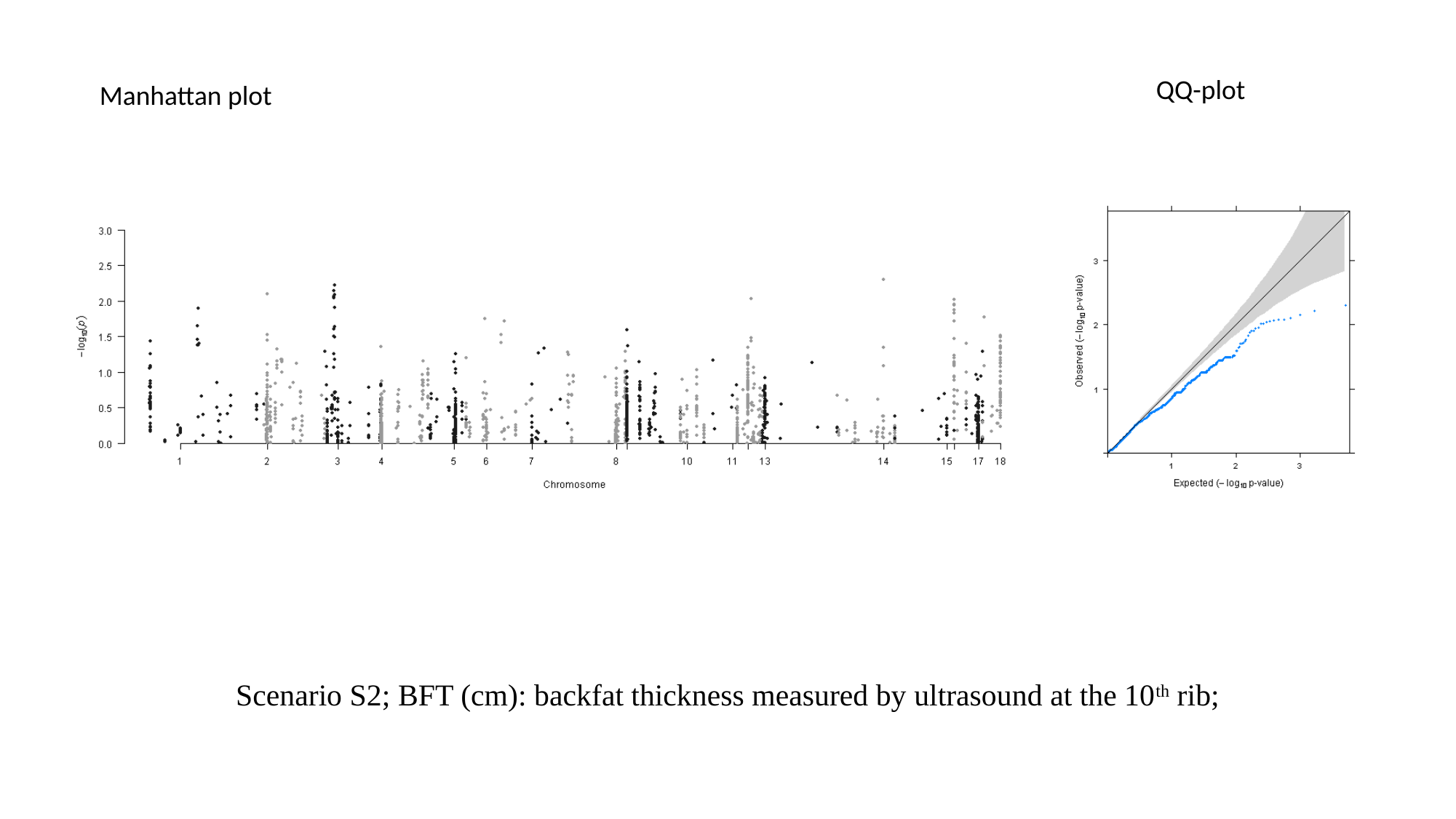

QQ-plot
Manhattan plot
# Scenario S2; BFT (cm): backfat thickness measured by ultrasound at the 10th rib;

## Slide 3
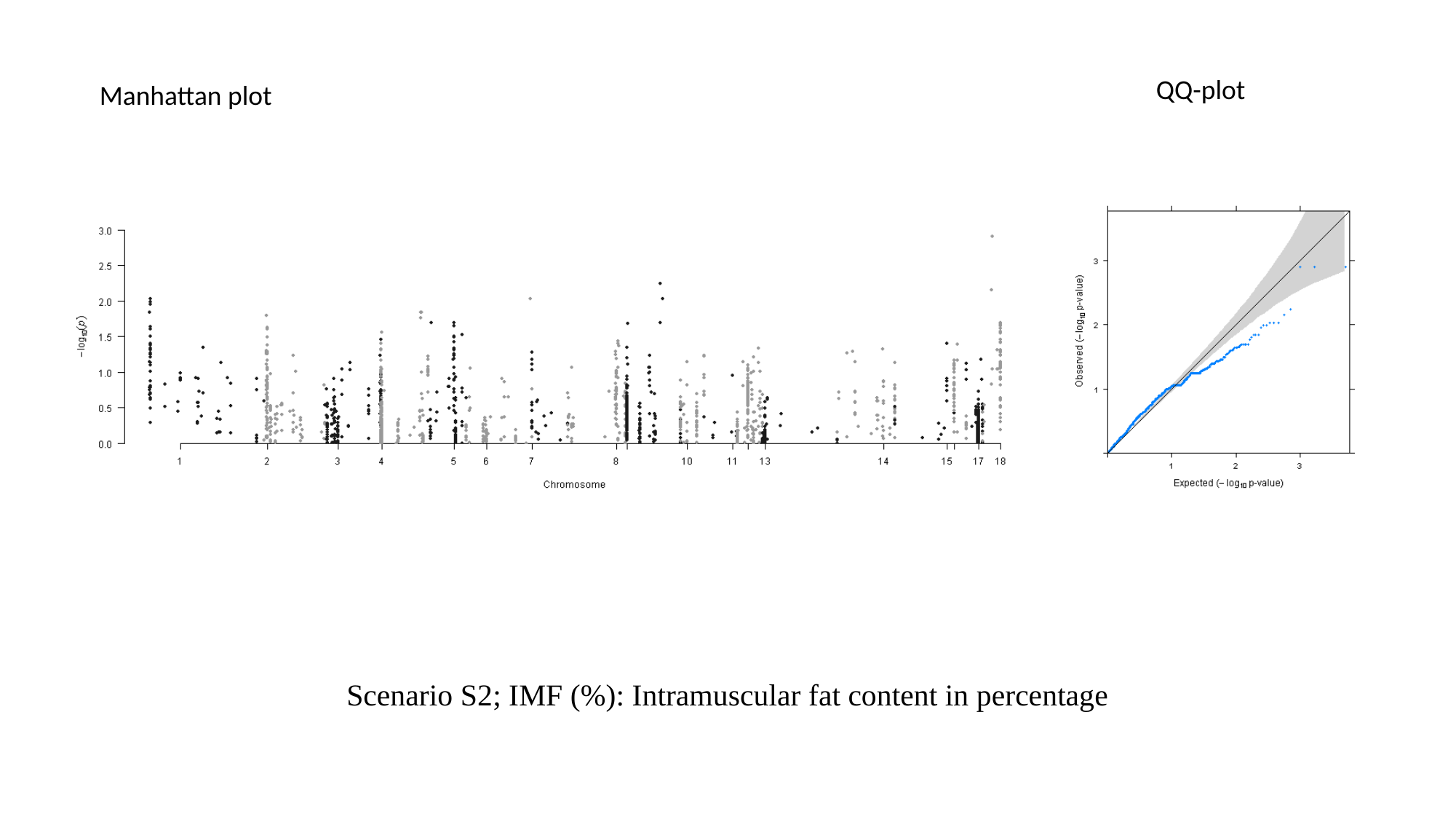

QQ-plot
Manhattan plot
# Scenario S2; IMF (%): Intramuscular fat content in percentage

## Slide 4
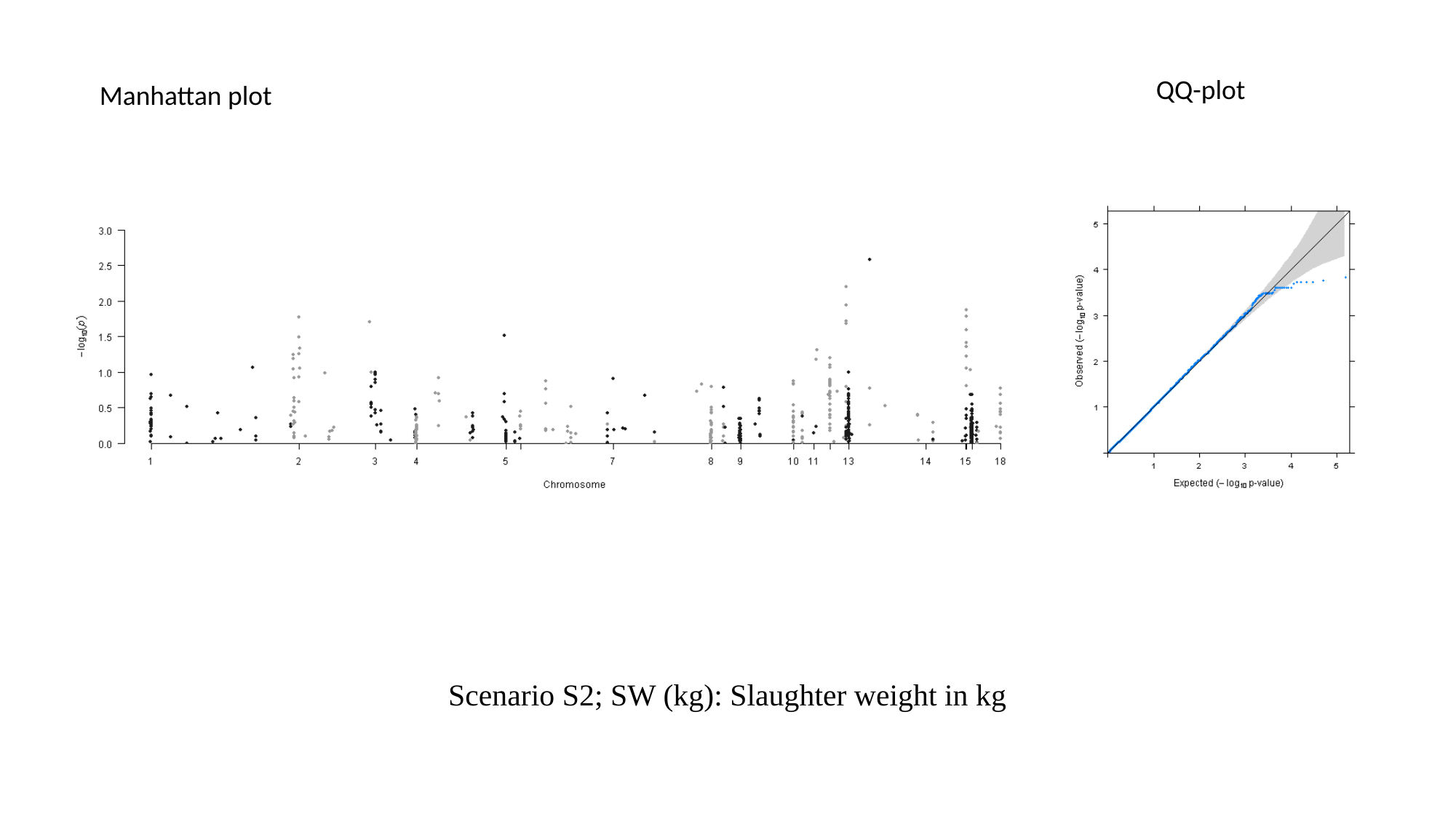

QQ-plot
Manhattan plot
# Scenario S2; SW (kg): Slaughter weight in kg

## Slide 5
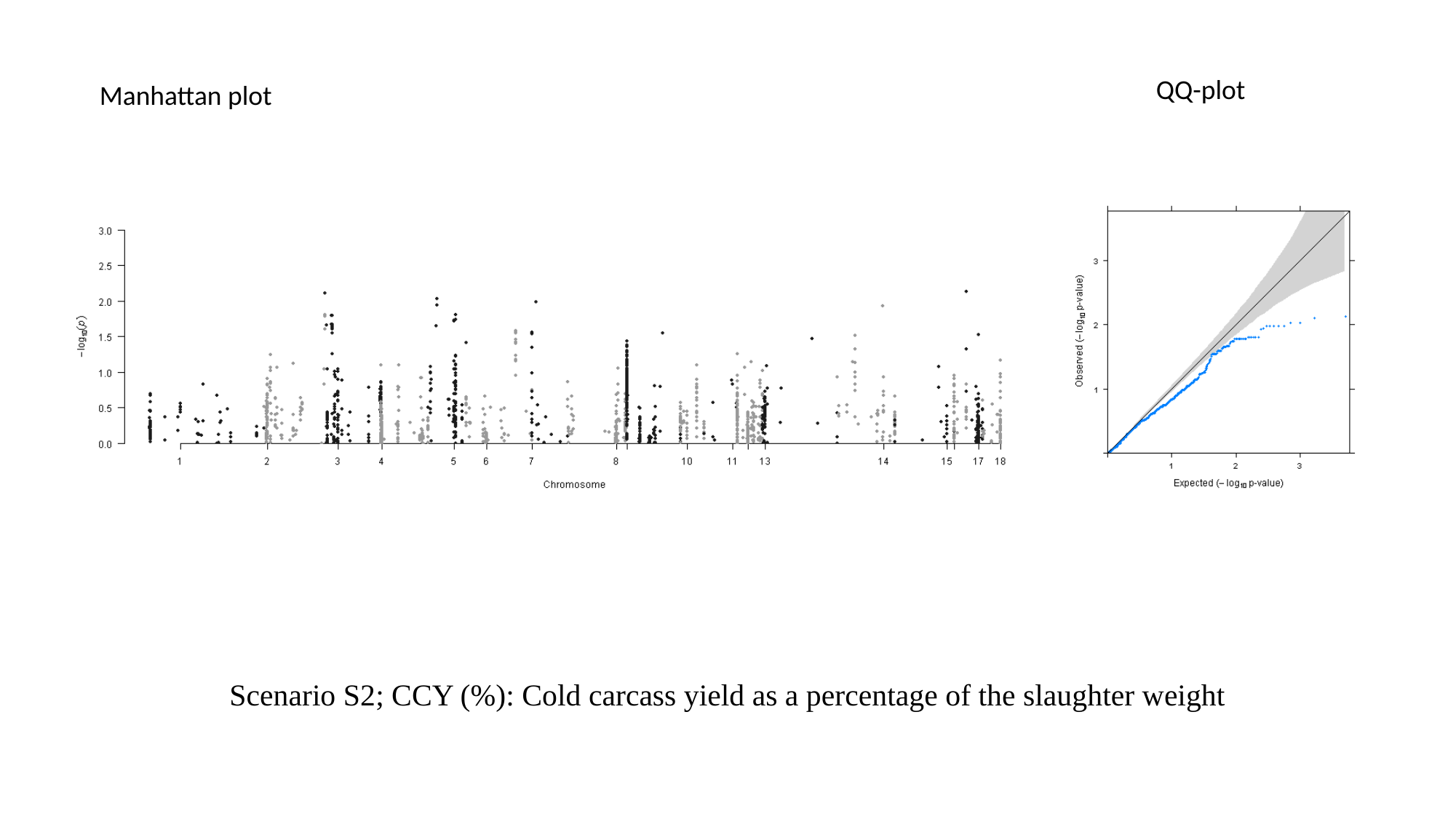

QQ-plot
Manhattan plot
# Scenario S2; CCY (%): Cold carcass yield as a percentage of the slaughter weight

## Slide 6
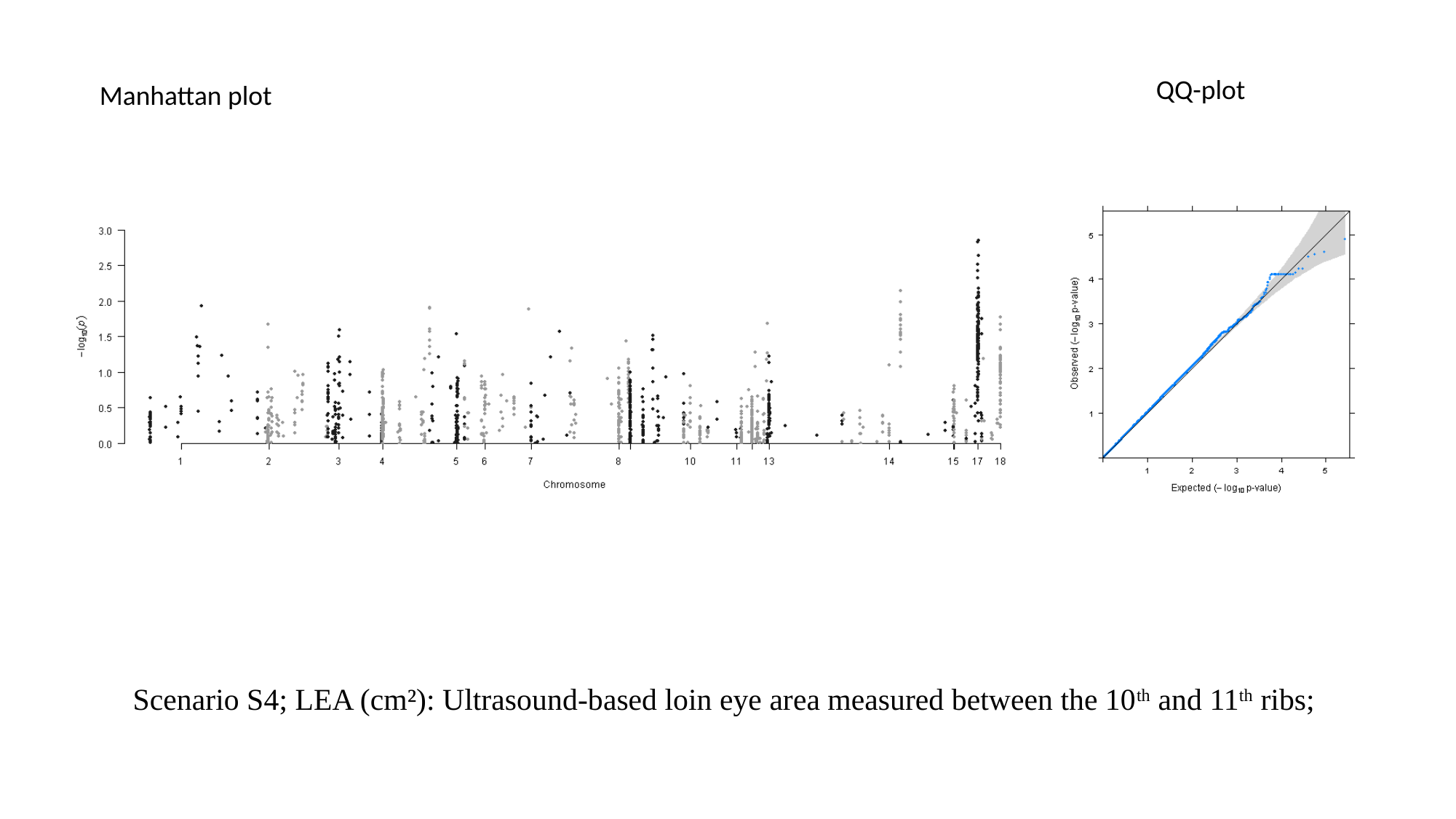

QQ-plot
Manhattan plot
# Scenario S4; LEA (cm²): Ultrasound-based loin eye area measured between the 10th and 11th ribs;

## Slide 7
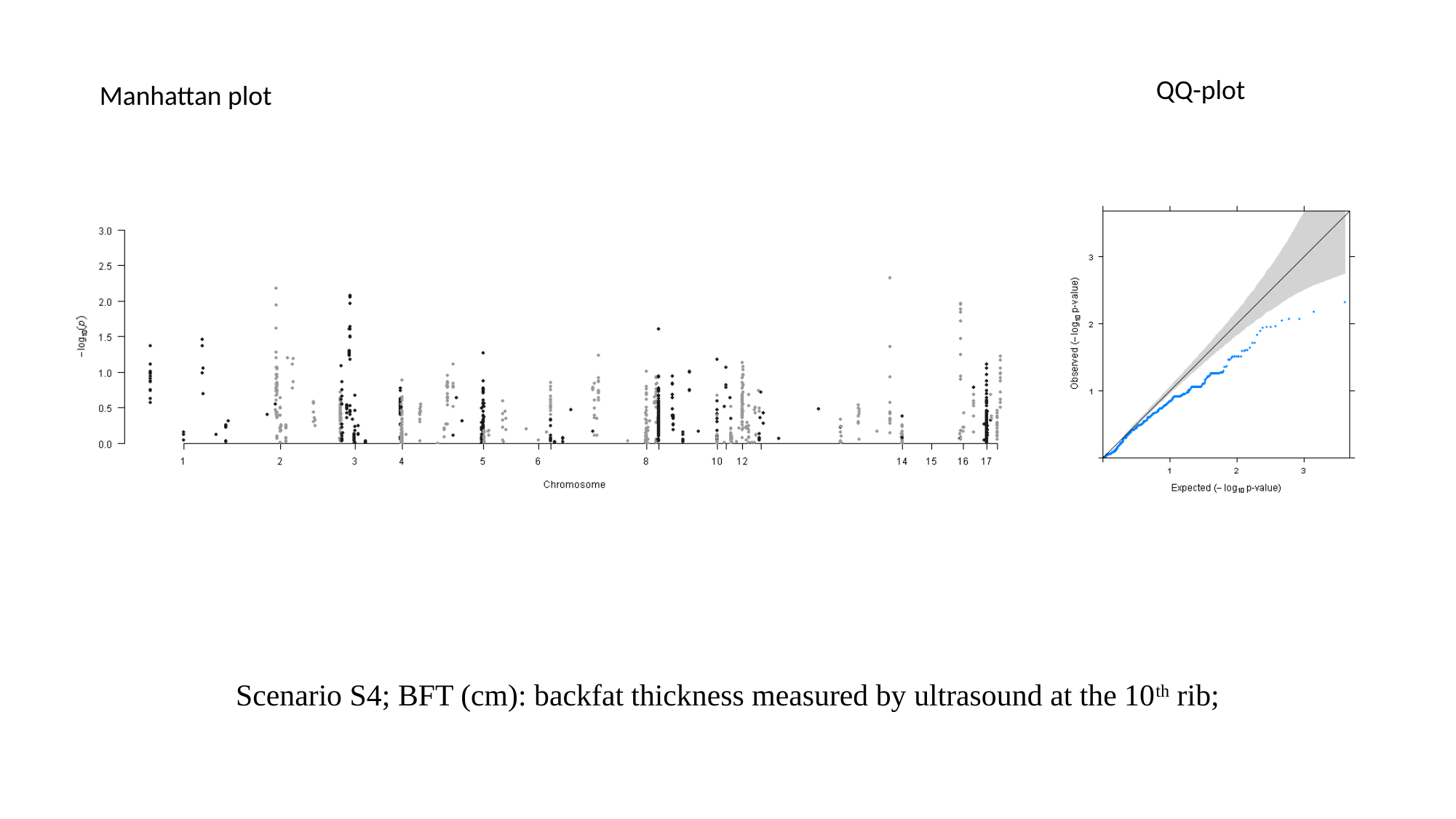

QQ-plot
Manhattan plot
# Scenario S4; BFT (cm): backfat thickness measured by ultrasound at the 10th rib;

## Slide 8
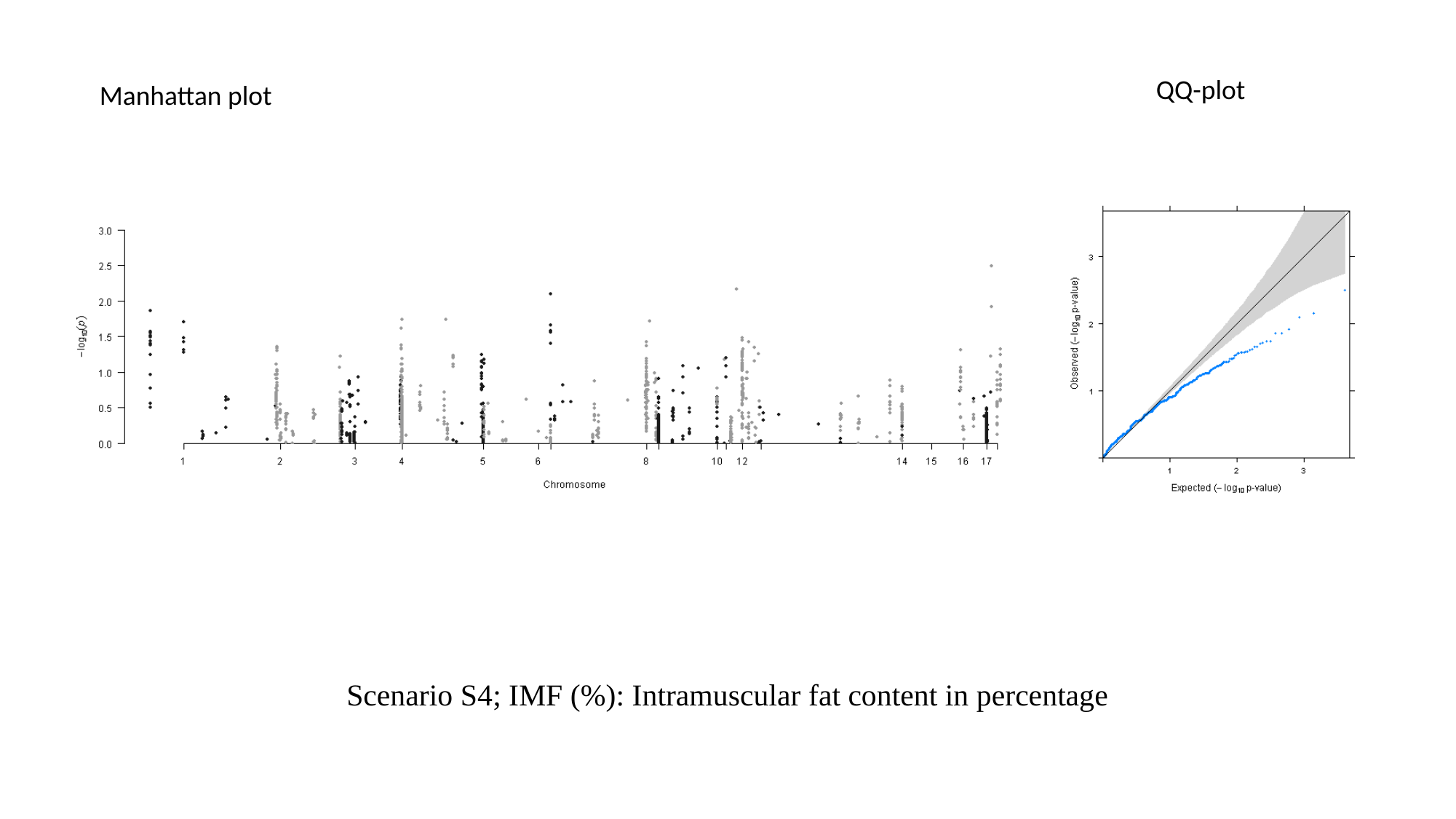

QQ-plot
Manhattan plot
# Scenario S4; IMF (%): Intramuscular fat content in percentage

## Slide 9
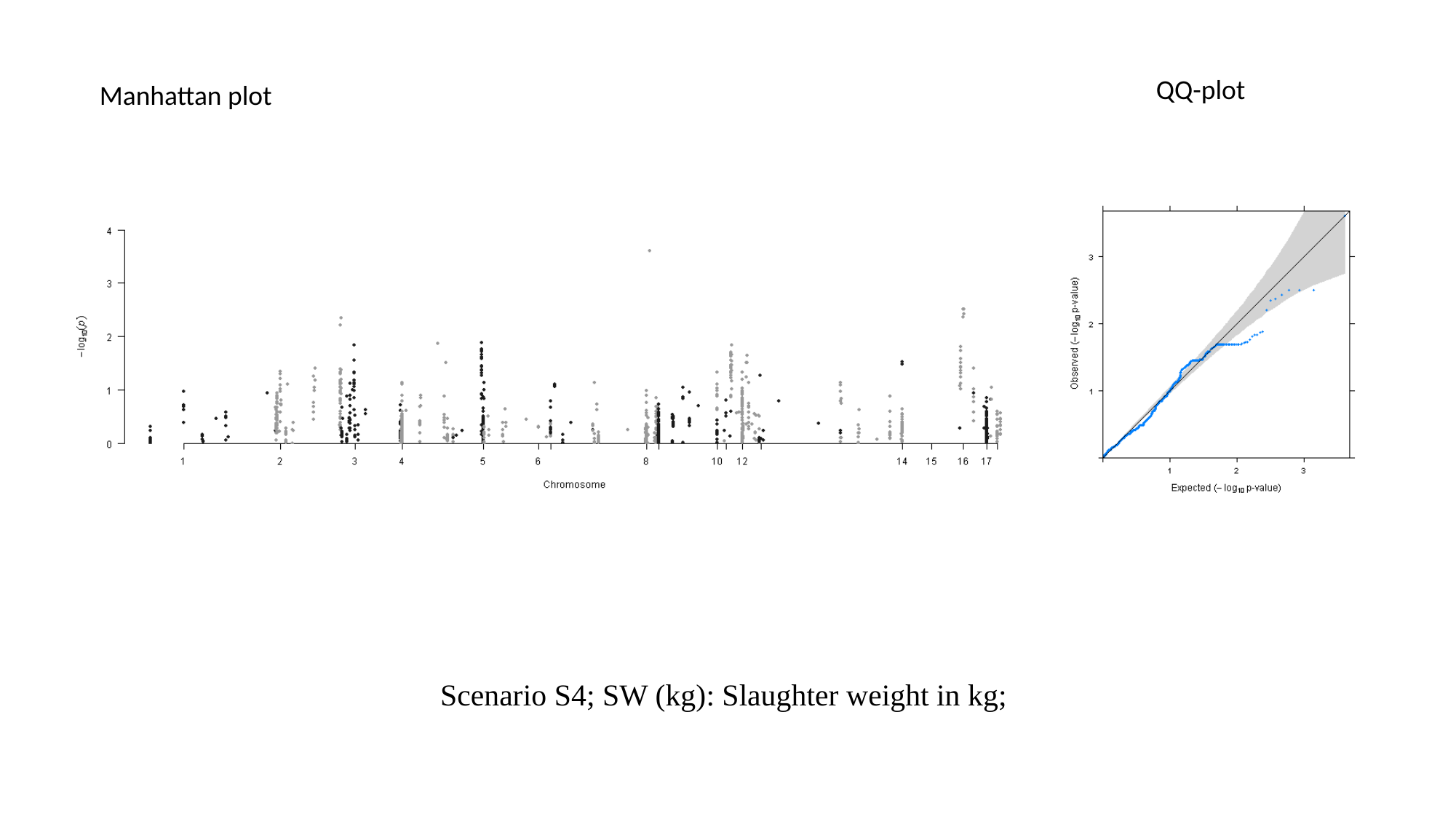

QQ-plot
Manhattan plot
# Scenario S4; SW (kg): Slaughter weight in kg;

## Slide 10
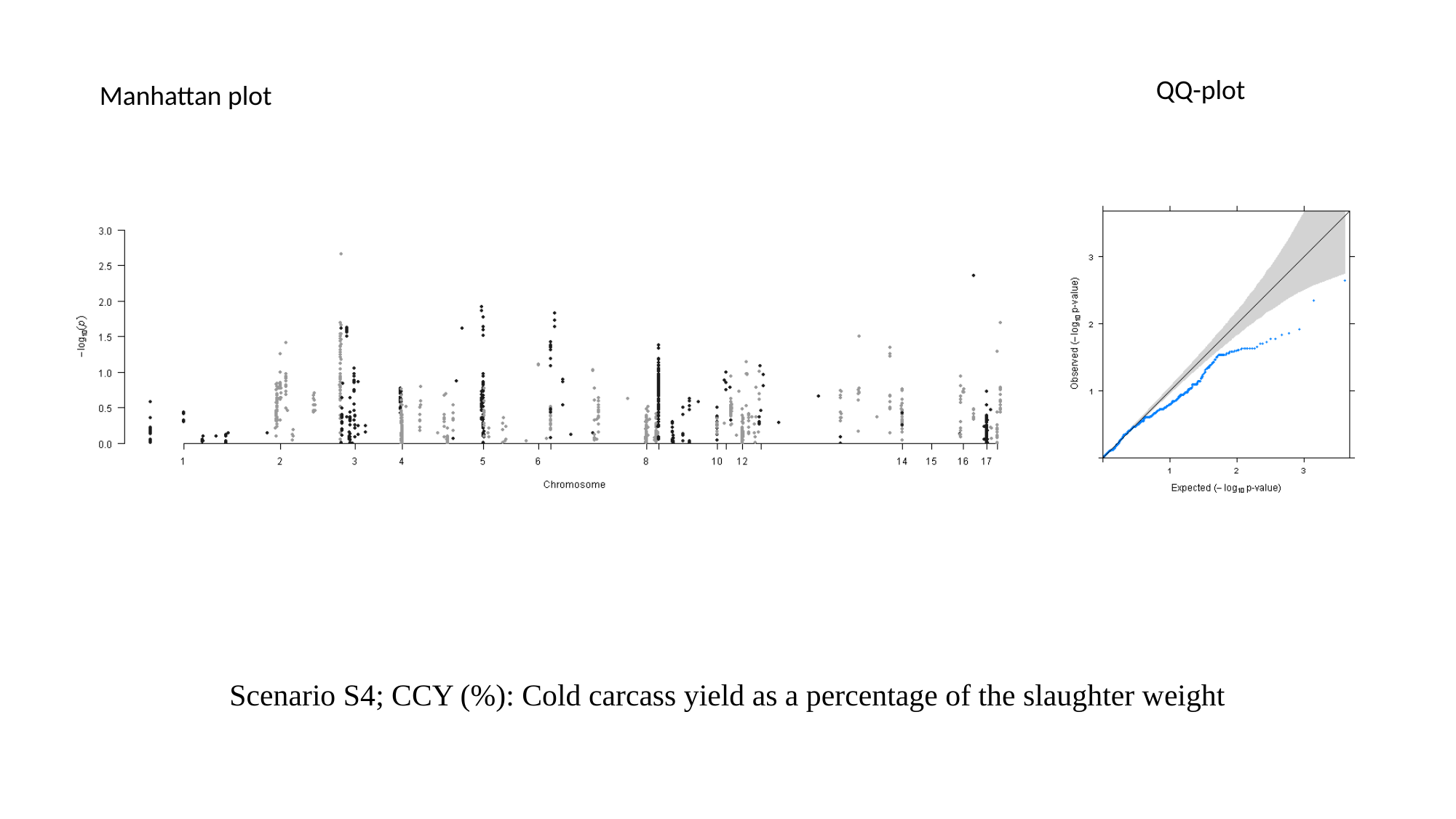

QQ-plot
Manhattan plot
# Scenario S4; CCY (%): Cold carcass yield as a percentage of the slaughter weight

## Slide 11
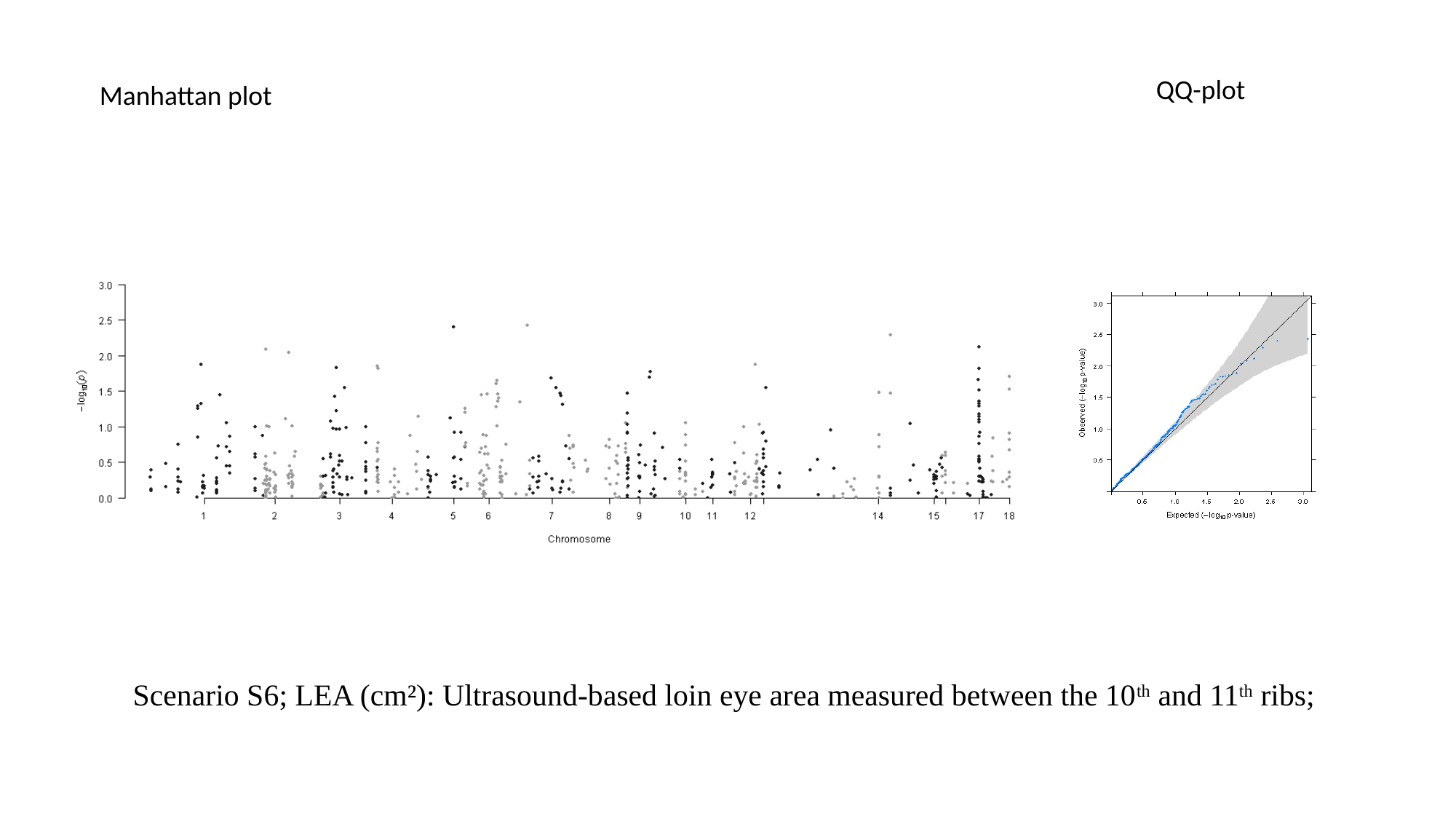

QQ-plot
Manhattan plot
# Scenario S6; LEA (cm²): Ultrasound-based loin eye area measured between the 10th and 11th ribs;

## Slide 12
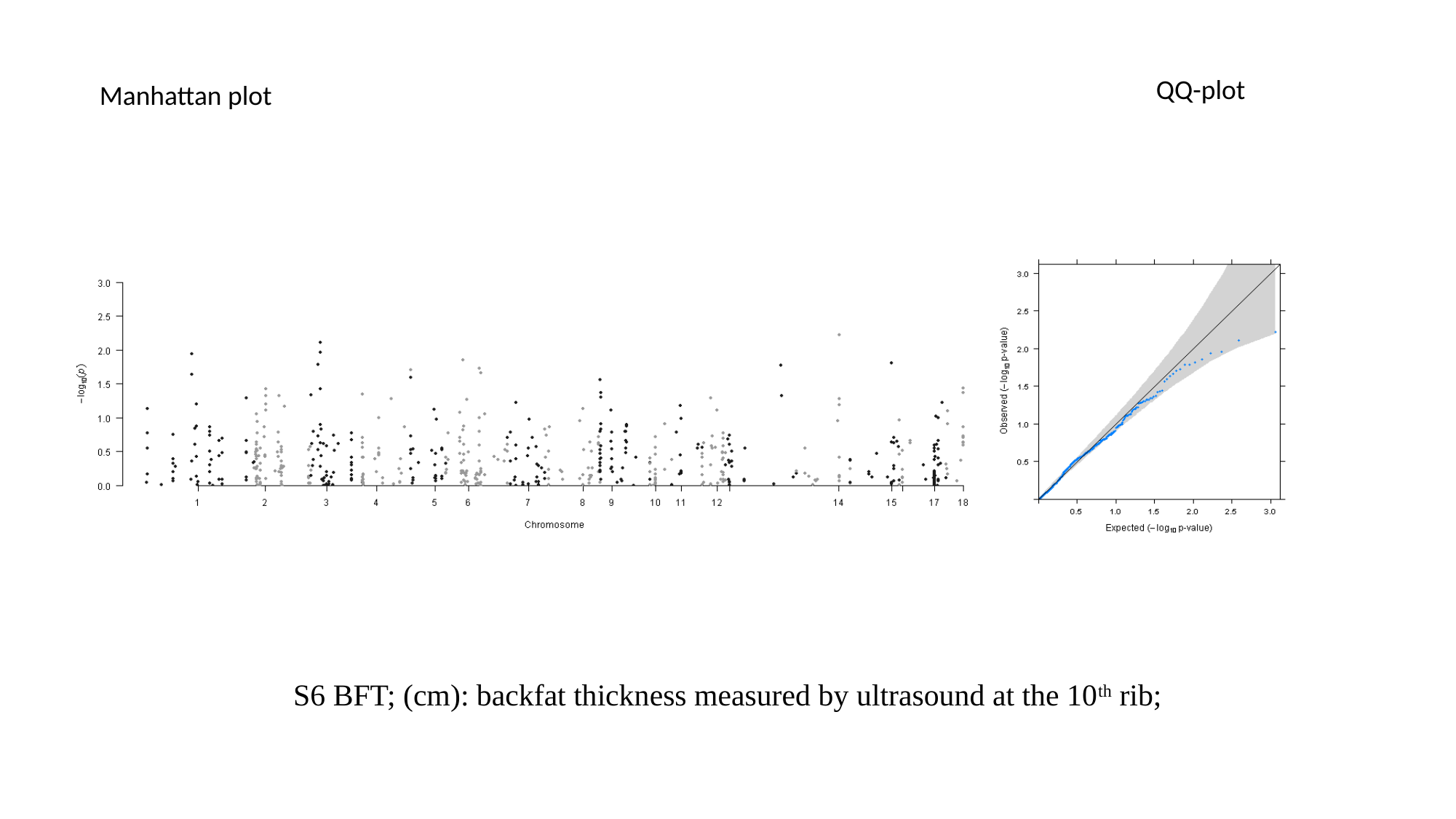

QQ-plot
Manhattan plot
# S6 BFT; (cm): backfat thickness measured by ultrasound at the 10th rib;

## Slide 13
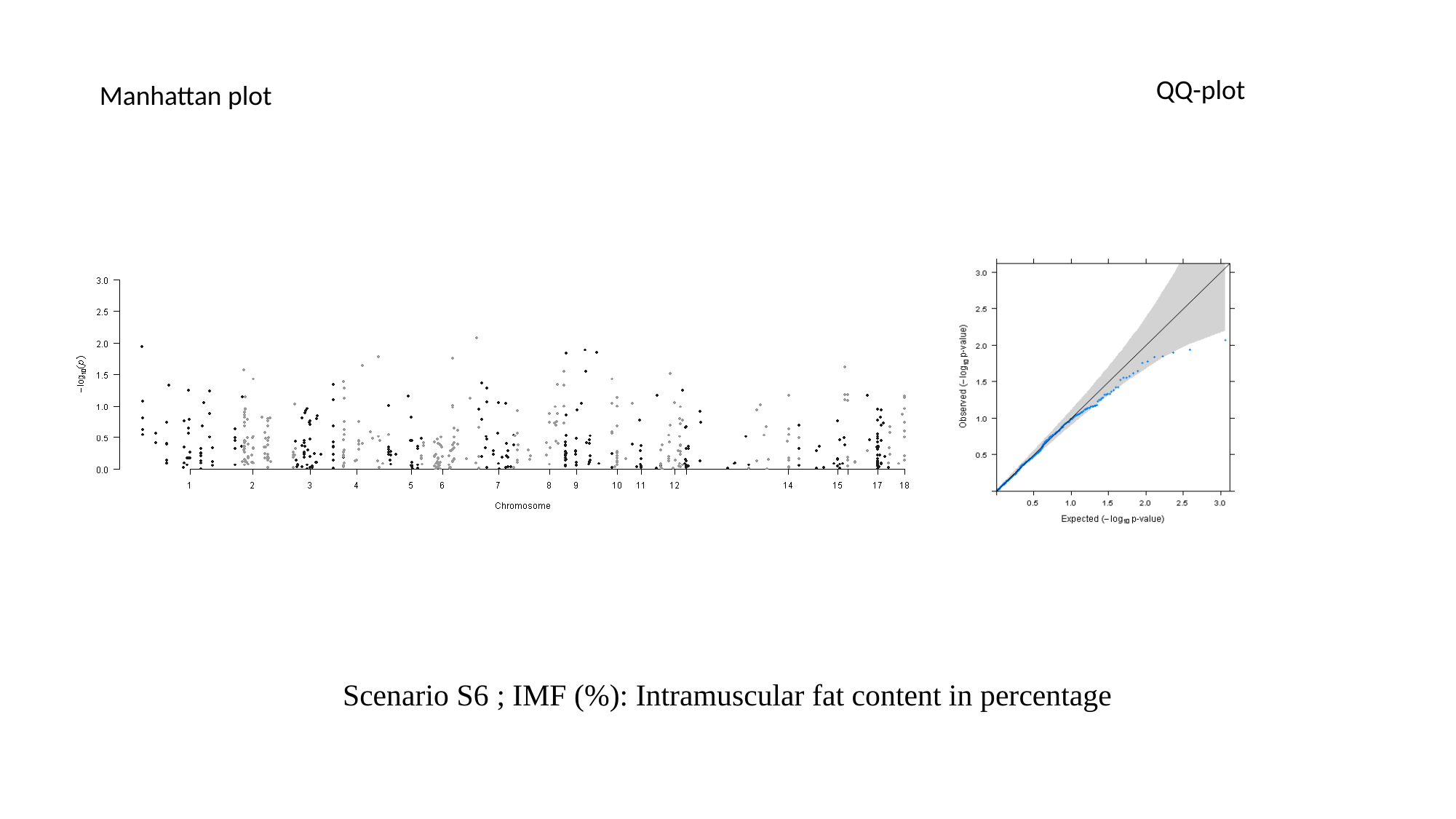

QQ-plot
Manhattan plot
# Scenario S6 ; IMF (%): Intramuscular fat content in percentage

## Slide 14
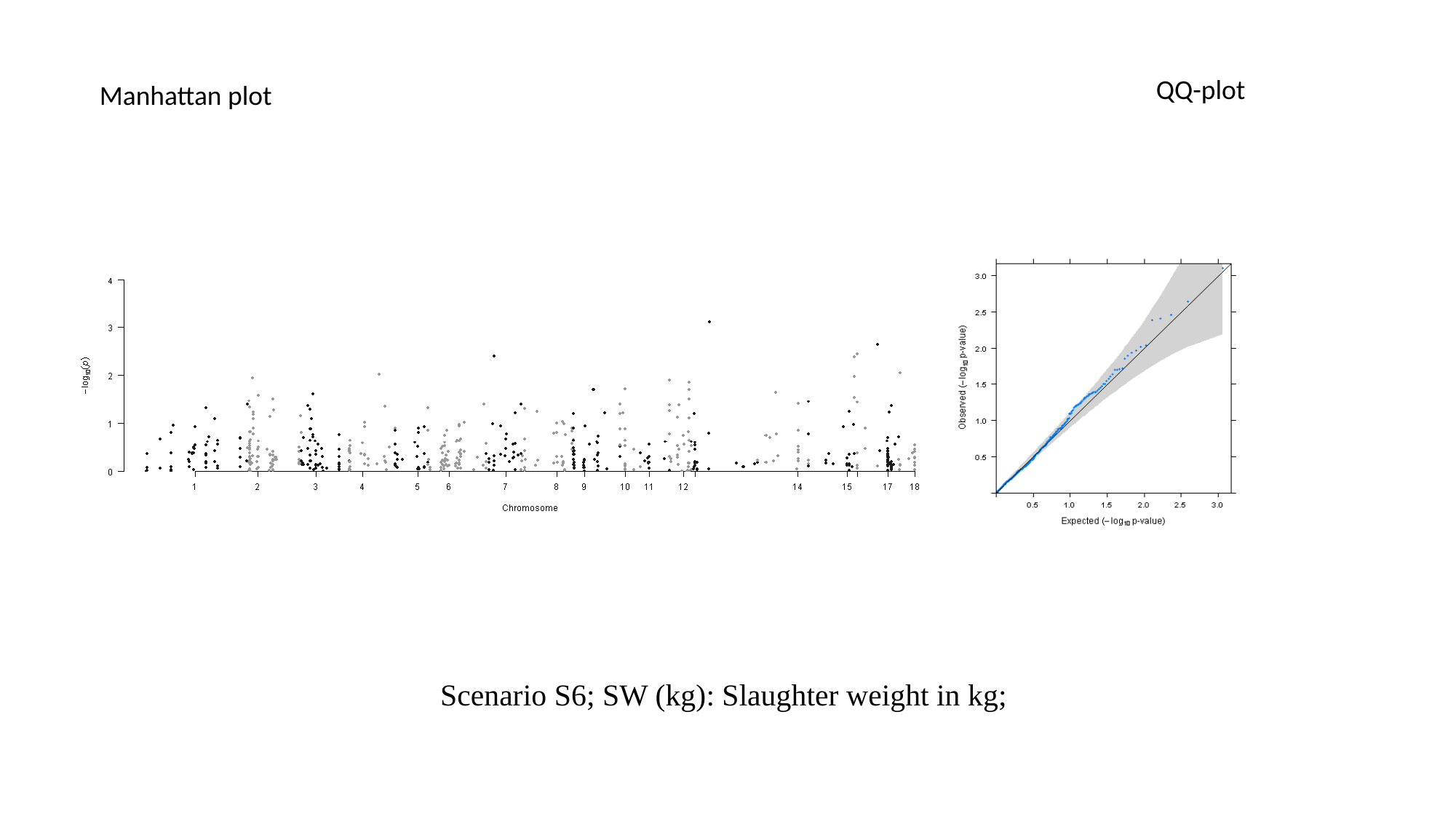

QQ-plot
Manhattan plot
# Scenario S6; SW (kg): Slaughter weight in kg;

## Slide 15
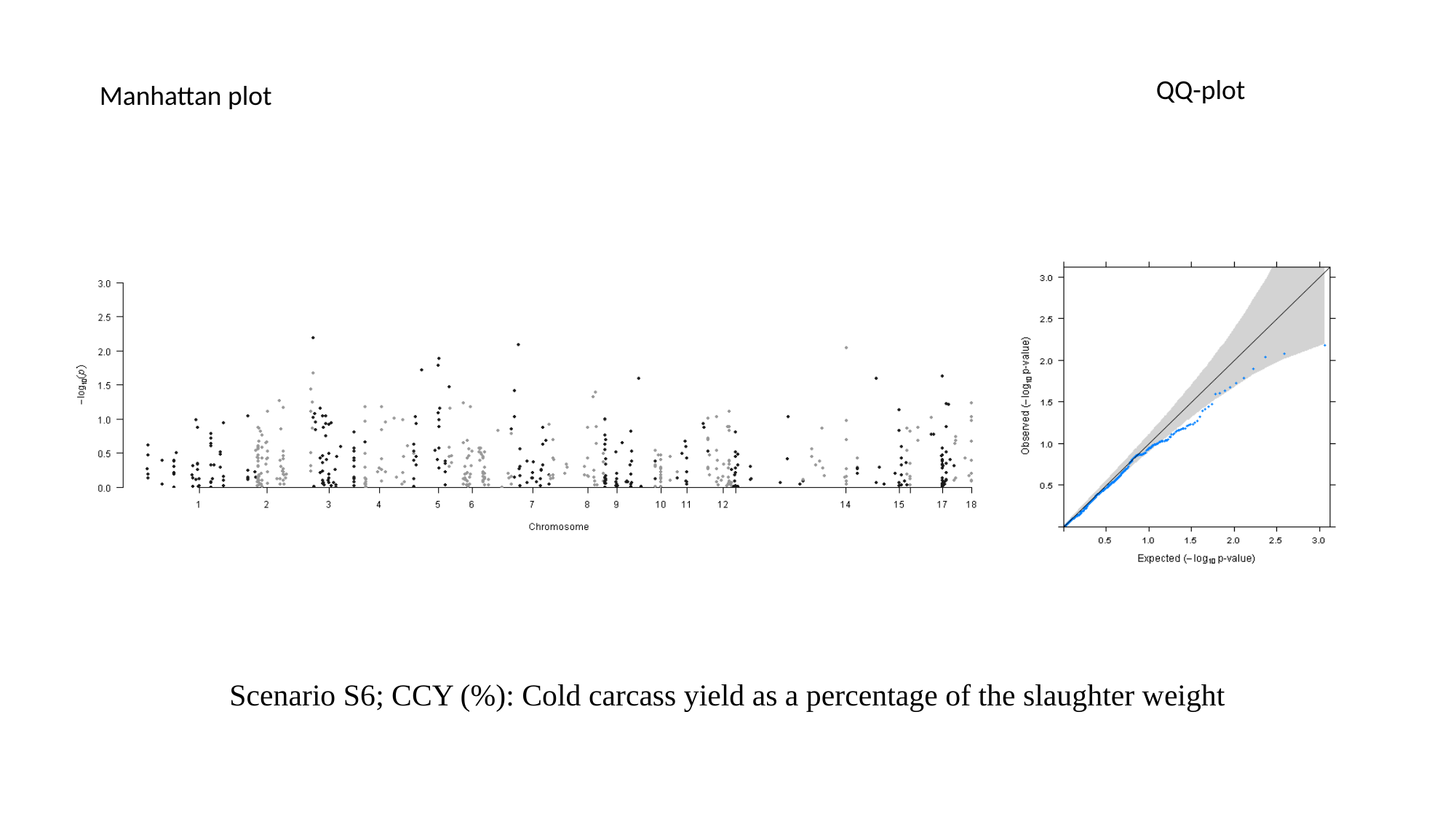

QQ-plot
Manhattan plot
# Scenario S6; CCY (%): Cold carcass yield as a percentage of the slaughter weight

## Slide 16
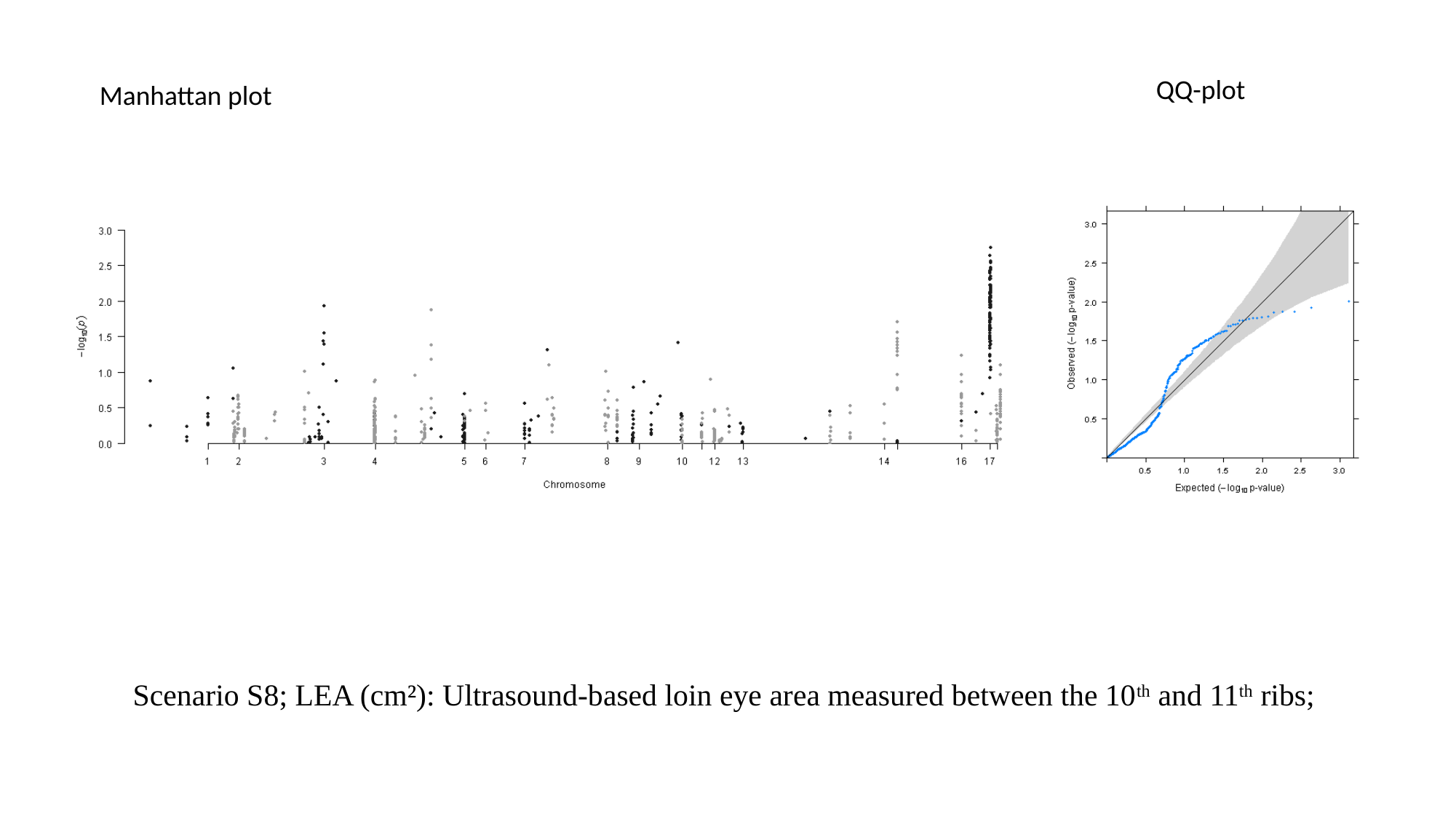

QQ-plot
Manhattan plot
# Scenario S8; LEA (cm²): Ultrasound-based loin eye area measured between the 10th and 11th ribs;

## Slide 17
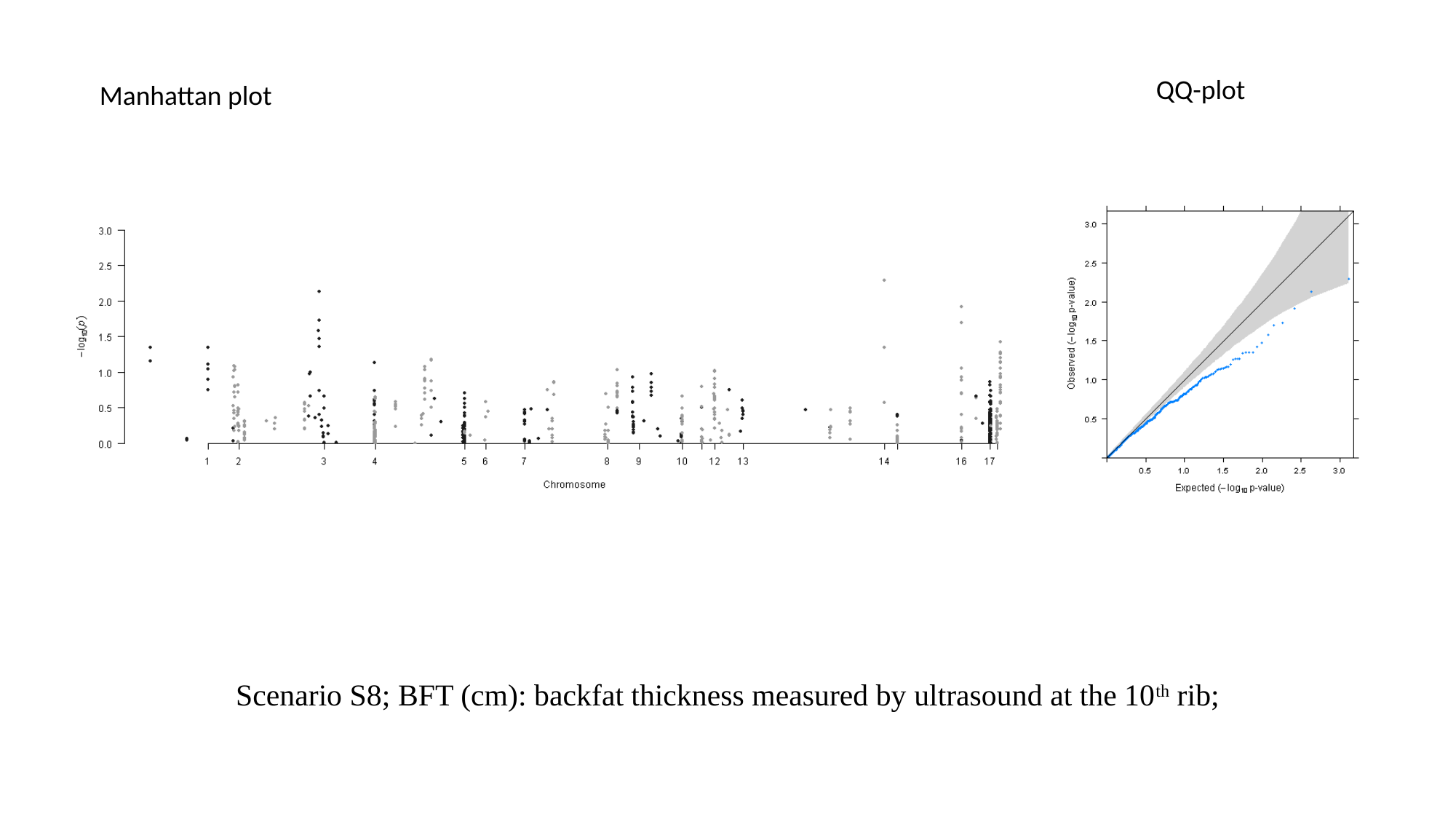

QQ-plot
Manhattan plot
# Scenario S8; BFT (cm): backfat thickness measured by ultrasound at the 10th rib;

## Slide 18
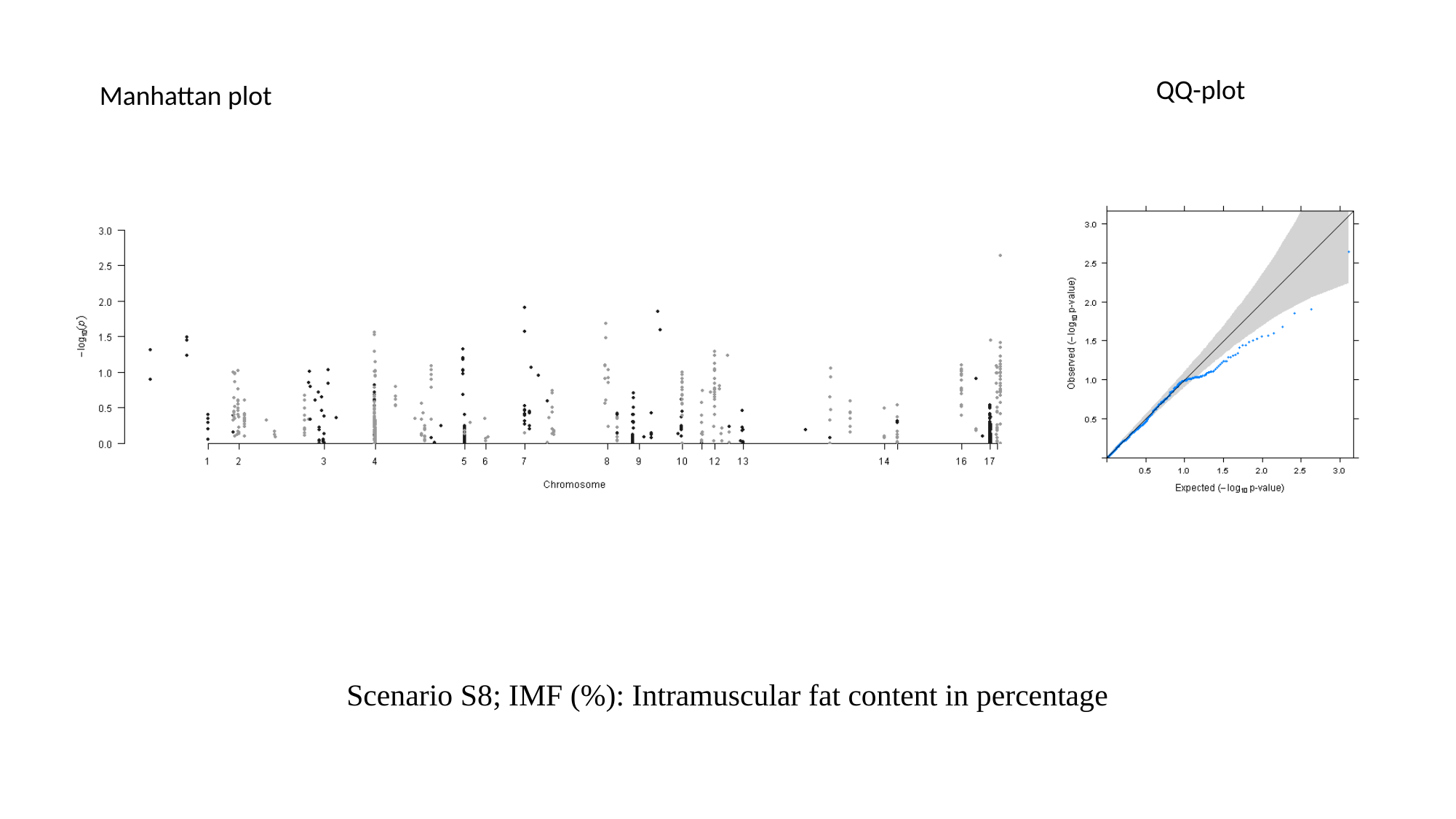

QQ-plot
Manhattan plot
# Scenario S8; IMF (%): Intramuscular fat content in percentage

## Slide 19
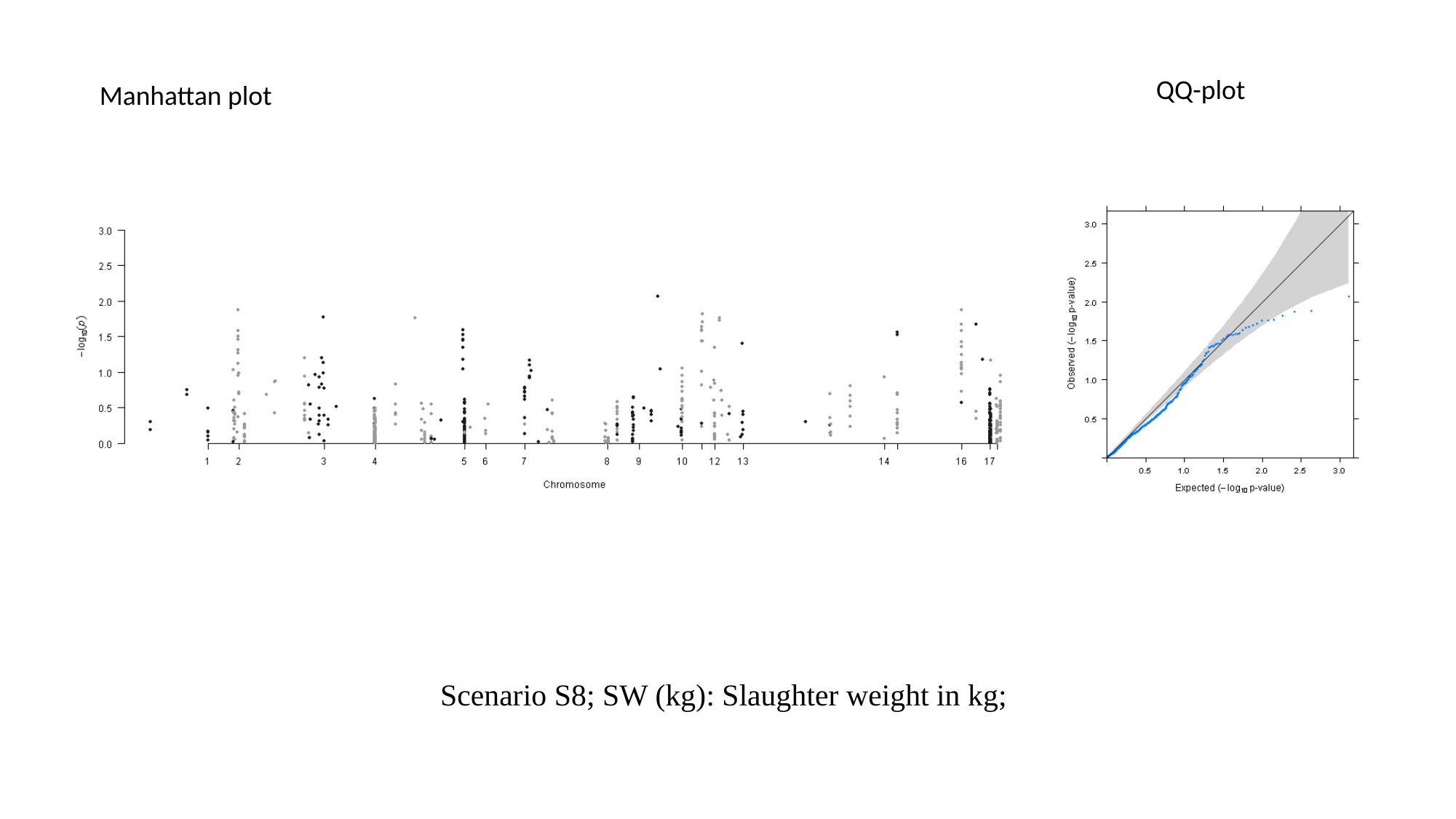

QQ-plot
Manhattan plot
# Scenario S8; SW (kg): Slaughter weight in kg;

## Slide 20
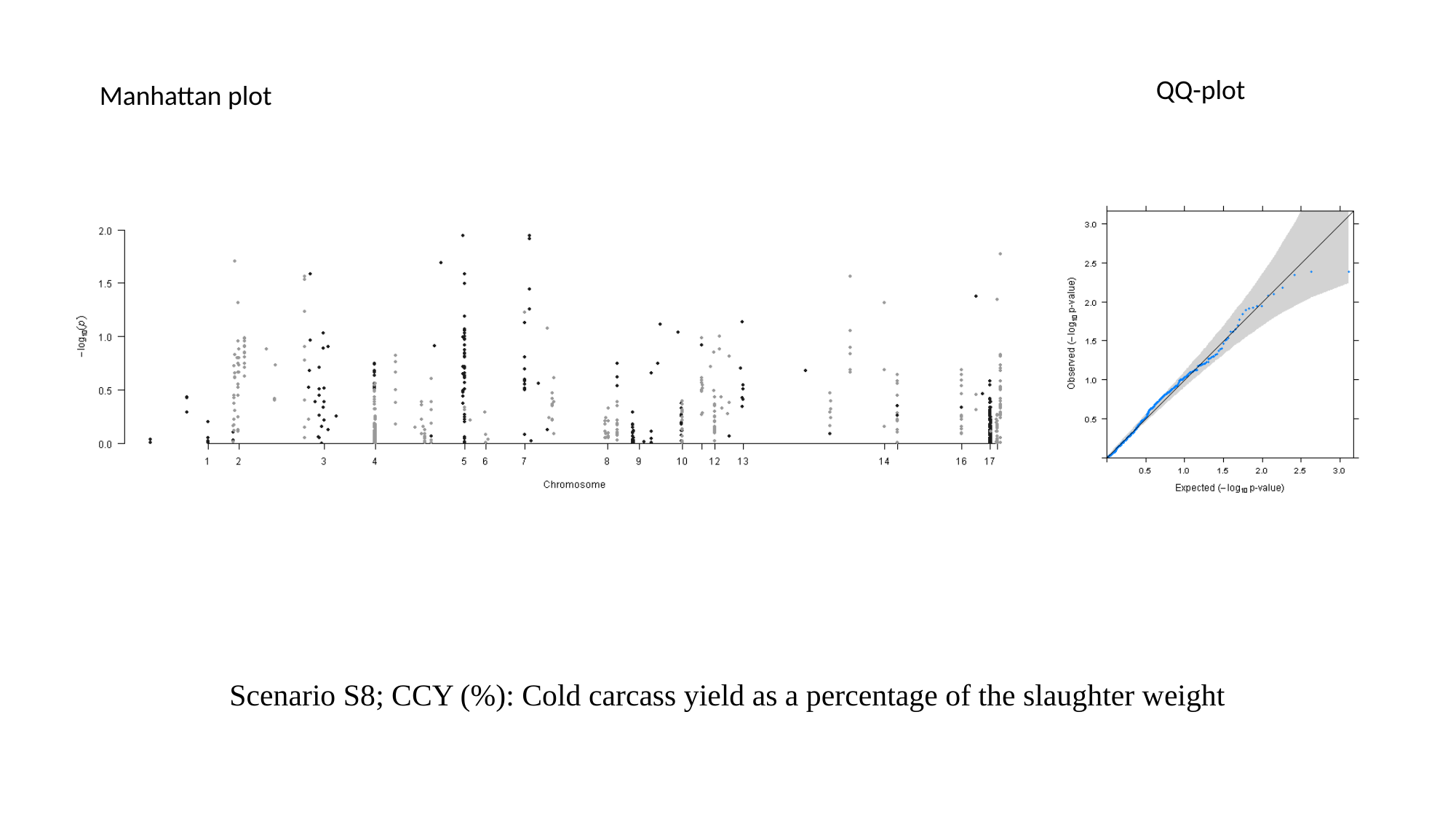

QQ-plot
Manhattan plot
# Scenario S8; CCY (%): Cold carcass yield as a percentage of the slaughter weight
